# A randomized trial of serological and cellular responses to hepatitis B vaccination in chronic kidney disease

**DOI:** 10.1371/journal.pone.0204477

**Published:** 2018-10-10

**Authors:** Elizabeth N. da Silva, Alan Baker, Jalila Alshekaili, Krishna Karpe, Matthew C. Cook

**Affiliations:** 1 Division of Immunology and Infection, John Curtin School of Medical Research, Australian National University, Canberra, ACT, Australia; 2 Translational Research Unit, Canberra Hospital, Garran, ACT, Australia; 3 Department of Immunology, Canberra Hospital, Garran, ACT, Australia; 4 Department of Immunology, Liverpool Hospital, Liverpool, NSW, Australia; 5 Department of Renal Medicine, The Canberra Hospital, Garran, ACT, Australia; Uniformed Services University, UNITED STATES

## Abstract

**Background:**

Chronic kidney disease (CKD) is associated with an increased risk of hepatitis B infection and impaired seroconversion to hepatitis B vaccine (HBV). Studies examining augmented vaccine schedules to enhance seroconversion have so far been inconclusive. Furthermore, the defects responsible for impaired vaccine immunity in CKD have not yet been identified.

**Methods:**

We studied serological and cellular responses to HBV in CKD to identify a defect in vaccine-induced cellular responses that could account for impaired seroconversion in CKD and clarify the effects of an augmented vaccine dose schedule. We compared these results with responses to seasonal influenza vaccination (Fluvax).

**Results:**

We found a clear benefit in rates and magnitude of seroconversion after an augmented 40mcg HBV dose schedule in CKD. This permitted comparison of responders and non-responders. Serological non-responders with CKD exhibited reduction in CXCR3+CCR6- CXCR5+ memory T cells at baseline. Unlike Fluvax, HBV elicited a poor plasmablast (PB) response. Both vaccinations induced activation of the CXCR3^+^CCR6^-^ CCR7^-^ subset of circulating T follicular helper cells (cTFH), although this response was impaired in CKD after HBV.

**Conclusions:**

CKD confers a specific T cell defect that contributes to the impaired seroconversion to HBV.

## Introduction

End-stage renal disease confers a state of chronic immune deficiency, which results in poor response to standard vaccination regimens, and increased risk of infection, including chronic viral infections that can increase risk of cancer [1]. The risk of hepatitis B virus infection is particularly significant due to increased exposure to the virus during dialysis, and an increased risk of chronic infection and mortality [2]. Current guidelines recommend routine vaccination of dialysis patients with three or four 40mcg doses of hepatitis B vaccine (HBV), however, there is no universal dosing recommendation for pre-dialysis CKD patients [2–4]. The Centers for Disease Control and Prevention recommends 20mcg of HBV intramuscularly at 0, 1, 6 months in pre-dialysis patients. A significant proportion of CKD patients vaccinated prior to the commencement of dialysis remain at risk of contracting hepatitis B virus due to impaired seroconversion and rapid decline of protective titers [5]. Several studies have sought to address the possible benefit of augmenting the pre-dialysis schedule to four 40mg doses, however the data are so far inconclusive [5–7].

Elucidating the mechanism of vaccine unresponsiveness could help to characterize the nature of immune deficiency in CKD. So far, various cellular defects in immunity have been identified, including quantitative and functional defects in lymphocyte compartments [8–11] secondary to increased activation and apoptosis [8,9,12]. B cell compartment defects include reduced absolute numbers of total, innate, naïve and memory B cells [12]. One possible explanation is reduced expression of B-cell activating factor of the TNF-family (BAFF)-receptor [10]. T cell compartment defects include low absolute counts of total [8], naïve and central memory T cells of both CD4+ and CD8+ subsets [8,11]. Some of these defects have been partly attributed to the metabolic derangements of uremia and hyperphosphatemia [11]. At the same time, there is an inverse relation between glomerular filtration rate and markers of inflammation [13]. Possible explanations include decreased cytokine clearance, increased gut permeability, periodontitis, and oxidative stress of uremia [14–16].

High-affinity antibody responses depend on interactions between T and B cells in specialized microanatomical sites called germinal centers (GC), located in secondary lymphoid organs [17]. T follicular helper cells (TFH) are a subset of CD4 T cells characterized by high level expression of CXCR5 and PD-1, and the capacity to secrete IL-21[18]. TFH provide help to B cells at priming and to centrocytes in GC that results in their differentiation into memory B cells and long-lived plasma cells. Memory B cells are thought to serve as precursors of long-lived plasma cells after re-encounter with antigen. Recently, progress has been made in monitoring these cellular events in peripheral blood. 5–8 days after booster parenteral vaccination PB can be detected in the peripheral circulation [19–21]. These PB are antigen-specific, predominantly of IgG isotype, and their peak in peripheral blood correlates with serological response [19,20,22]. Similarly, a peripheral cellular signature of events within GC has been pursued by enumerating putative circulating counterparts of TFH [23–26]. These cells are memory CD4^+^ T cells expressing the B-cell-zone homing chemokine receptor (CXCR)-5, high levels of Inducible Co-stimulator (ICOS) [23] which is critical for TFH development [27], and Programmed Cell Death (PD)-1 [28], a marker of recent T cell activation via the T cell receptor. Chemokine receptors CXCR3 and CCR6 have previously been identified as surrogate markers of effector T cell differentiation including within GC (CXCR3^+^CCR6^-^ (Th1), CXCR3^-^CCR6^-^ (Th2) and CXCR3^-^CCR6^+^(Th17)) [29,30] and have also been demonstrated to distinguish functional cTFH subsets [24]. cTFH that express CXCR3 but not CCR6 transiently increase 7 days following influenza vaccination, are antigen-specific, upregulate expression of ICOS and PD-1, and correlate with both serological response and the day 7 peak in PB [23].

We applied this approach to investigate the cellular events that accompany the serological response to immunization in CKD. We show that while primary immunization with HBV elicits a poor PB response, it specifically activates the CXCR3+CCR6-PD-1+CCR7- subset of cTFH in healthy age-matched controls (HC). This cTFH response is not seen in CKD. By contrast, Fluvax generates robust PB and cTFH responses in both CKD and HC. Additionally, CKD patients who fail to sero-respond to HBV demonstrate reduced proportions of CXCR3+CCR6- cTFH at baseline prior to vaccination. Finally, we show that an augmented 40mcg dose schedule of HBV can overcome the serological defect in many CKD patients, although this does not restore the peripheral cellular events to normality.

## Methods

### Patients

The study was approved by The Canberra Hospital Human Research and Ethics Committee in 2011. All recruits provided written informed consent to participate. Patients with stage 4 (15–29 ml/min/1.73m^2^) or 5 (<15 ml/min/1.73m^2^) CKD requiring HBV for routine care were recruited through the Department of Renal Medicine at The Canberra Hospital. The study protocol was modified and approved in 2013 to include recruitment of patients with stage 4 or 5 CKD requiring routine Fluvax, and HC requiring routine vaccination with either HBV or Fluvax. HC requiring routine HBV or Fluvax for occupational reasons were recruited via the Occupation Medicine Unit at the Canberra Hospital, and via a private geriatrics outpatient clinic at National Capital Private Hospital, Canberra. Undetectable HBsAb titer (<10mIU/ml, ARCHITECT anti-HBs, Abbott, Ireland) was a pre-requisite to enrolment to the HBV arm. Trivalent, split virion, inactivated influenza vaccine (Fluvax containing A/California/7/2009, A/Victoria/361/2011 and B/Wisconsin/60/2008, CSL Australia) was used throughout the study period from 2012–2013. HC received standard schedule [18] HBV (recombinant hepatitis B surface antigen, Engerix-B, GlaxoSmithKline) consisting of three doses of 20mcg, at 0, 1 and 4 months or at 0, 1 and 6 months. CKD patients receiving HBV were randomized, at a ratio of 1:1, via computer generated random number (https://www.randomizer.org) to receive either four doses of 20mcg or four doses of 40mcg of HBV at 0, 1, 2, and 6 months. Consecutively numbered, opaque, sealed envelopes were opened in sequence at the time of randomization. Patients were not blinded to their randomization because the augmented dose required two intramuscular injections instead of one, and this was detailed in the patient information sheet read by patients prior to study enrolment. Exclusion criteria for CKD patients were dialysis or kidney transplant. Exclusion criteria for all groups included cancer, infection, chronic inflammatory disease, immunosuppressive medication or other defined immunodeficiency.

### Serological analysis

All subjects who received HBV underwent serum collection four weeks after the final dose for HBsAb titer, with seroconversion defined as a titer ≥ 10 mIU/ml. The study protocol was modified and approved in 2015 to include collection of longitudinal serology from CKD patients and HC already recruited to the study. Serum collection was repeated between 3–5 years later to determine longitudinal HBsAb titer for both CKD patients and healthy controls. Both patients and healthy controls were recruited between January 2011 and July 2018, however only patients recruited by the end of December 2015, and who completed all follow-up by December 2016, have been included in the current analysis. The authors confirm that trials for this intervention are registered with the Australian New Zealand Clinical Trial Registry (ACTRN12618000631202).

### Sample collection

Peripheral blood was collected before (median 0, range -29-0, mean 1.5 days) and approximately 7 days after (median 7, range 6–13, mean 7.5 days) either Fluvax or the first dose of HBV. PBMCs were separated by density gradient centrifugation on Ficoll (Ficoll-Paque PLUS, GE Healthcare), then washed and frozen for batched analysis.

### Lymphocyte analysis with flow cytometry

Samples were thawed and analyzed by FACS in batches, ensuring paired samples (baseline and day 7) for any given patient were analyzed in the same batch. Samples were labelled using anti-CD3 PerCP, CD14 PerCP, CD19 PE-Cy7, CD38 V450, CD27 APC, HLA-DR APC-H7, followed by fixation and permeabilization (Cytofix/Cytoperm, BD biosciences), then labelled for intracellular immunoglobulin expression using anti-IgG PE, IgA biotin and IgM FITC. Cells were then stained with streptavidin-conjugated V500 in permeabilization-compatible wash, before suspension in phosphate buffered saline (PBS) with 2% fetal bovine serum (FBS)(GIBCO, LifeTechnologies). (All conjugated antibodies were obtained from BD biosciences except IgM FITC (Dako)). In separate experiments (as indicated), samples were stained for cTFH using anti-CD3 FITC, CCR6 PE, CCR7 PE-Cy7 (BD biosciences), CD4 APC-Cy7, CD45RA Pacific Blue, CXCR5 PerCP/Cy5.5, CXCR3 Brilliant Violet 510 (Biolegend), PD-1 biotin and streptavidin-APC (eBioscience). After staining, samples were washed in (PBS) with 2%(FBS), and fixed (Cytofix, BD biosciences). Samples were re-suspended in PBS for data acquisition on FACSCanto II or Fortessa (BD Biosciences). FACS data were analyzed using FlowJo (Version 10, Treestar Inc). Randomly selected samples underwent repeat analyses using either panel, to determine assay reproducibility and validity. To confirm the validity of the assay on frozen and thawed samples, paired analyses were performed on fresh and frozen samples from a single donor (data not shown).

### Lymphocyte assays

Routine blood samples were collected at baseline from patients and controls enrolled in the HBV arm, for full blood count and lymphocyte subsets using dual platform analysis (Beckman Coulter LH750 Hematology Analyzer and FacsCantoII, BD Biosciences), and serum immunoglobulins (Architect, Abbott). Absolute lymphocyte counts were used in conjunction with experimental T cell panel analyses to calculate memory T cell parameters.

### Statistical methods

Statistical analyses were performed using Prism (Version 6; GraphPad Software Inc). The null hypothesis that samples from one experimental group were equally likely to be greater or less than samples from the other group were tested using the Mann-Whitney U test. In experiments where more than one hypothesis was tested, p values were adjusted using Bonferroni correction of a type I error set to ≤0.05 (unless stated otherwise). Rank correlation was tested using Spearman correlation coefficient. Sex composition of each treatment group was analysed using Fisher’s exact test.

## Results

### Enrolment

**Fig 1:**
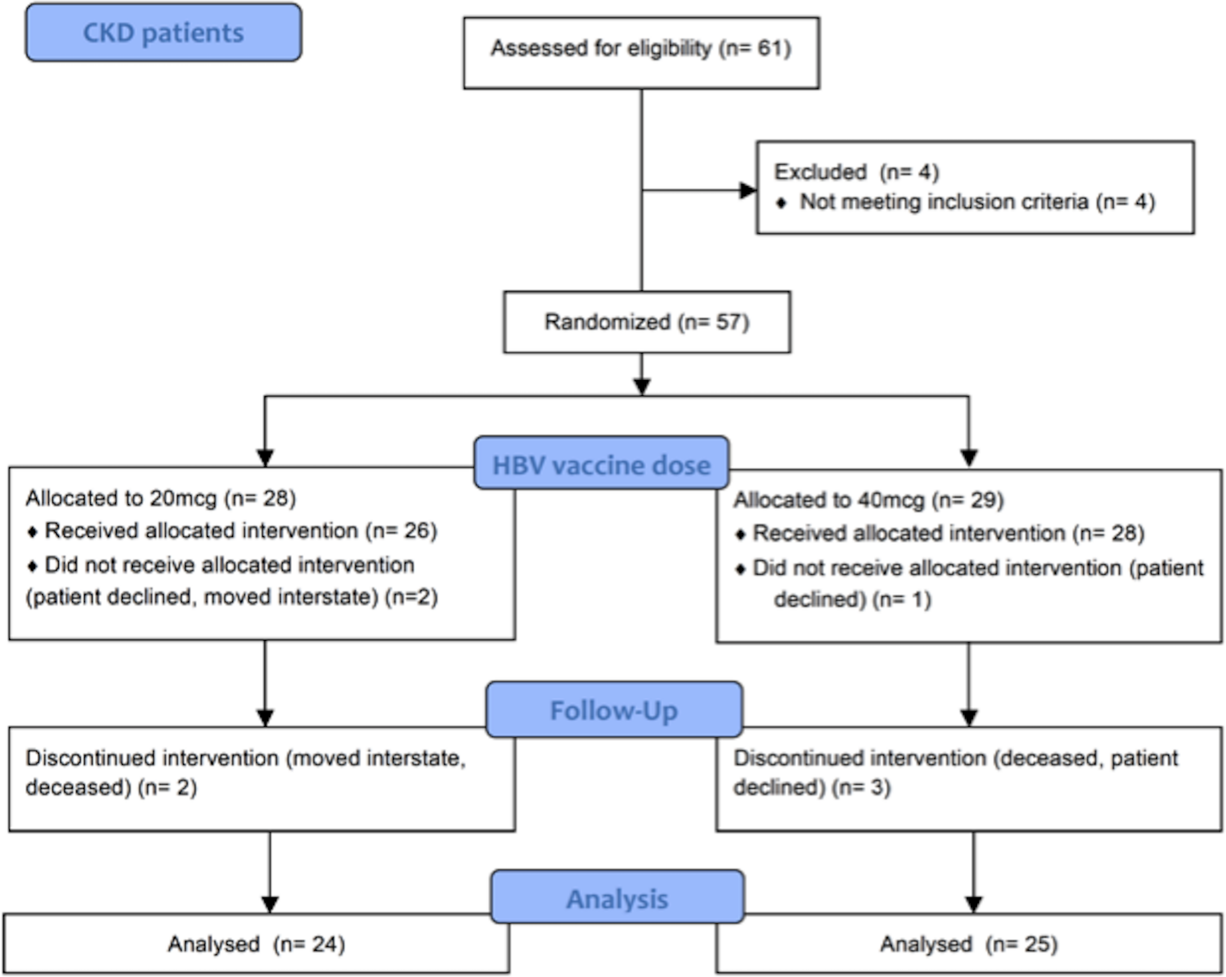
Flow chart of enrolment of CKD patients and randomization to receive primary hepatitis B vaccination with either four doses of 20mcg or 40mcg.

Fifty-seven patients with stage 4 or 5 CKD were enrolled between 2011 and 2015, inclusive (Fig 1). Patient demographics and characteristics are shown in Table 1. 22 patients had diabetic nephropathy, of which 18 were taking insulin, 7 of whom were also on oral hypoglycaemic medication, and three were taking oral hypoglycemic medication only. Only one patient with diabetic nephropathy was not taking either medication. Other causes of renal disease were membranous glomerulonephritis (4), IgA nephropathy (4), renovascular disease (4), vasculitis (2), focal segmental glomerulosclerosis (2), mesangiocapilliary glomerulonephritis (1), lupus nephritis (1), hypertension (1), polycystic kidney disease (1), embolic disease (1), obstructive uropathy (1), and unknown cause (13). Of these patients, two were taking insulin and oral hypoglycaemic medication (both with unknown cause of renal disease), and three were taking oral hypoglycaemic medication only (two patients with IgA nephropathy, and one with renovascular disease). Sample collection is summarized in S1 Fig.

**Table 1:** Patients demographics according to vaccine groups.

	CKD	HC	p value
**Hepatitis B vaccine**			
Patients (n)	57	12	
Age (median, years)	69	53	0.1390*
Age (range, years)	34–85	21–93	
Male: Female	44:13	3:9	0.0010^#^
**Seasonal influenza vaccine**			
Patients (n)	6	17	
Age (median, years)	60.5	53	0.1615*
Age (range, years)	27–78	26–76	
Male: Female	5:1	9:8	0.3401^#^

CKD, chronic kidney disease; HC, healthy controls.

*Mann-Whitney U test

#Fisher’s exact test.

### Lymphocyte subset analysis

**Fig 2:**
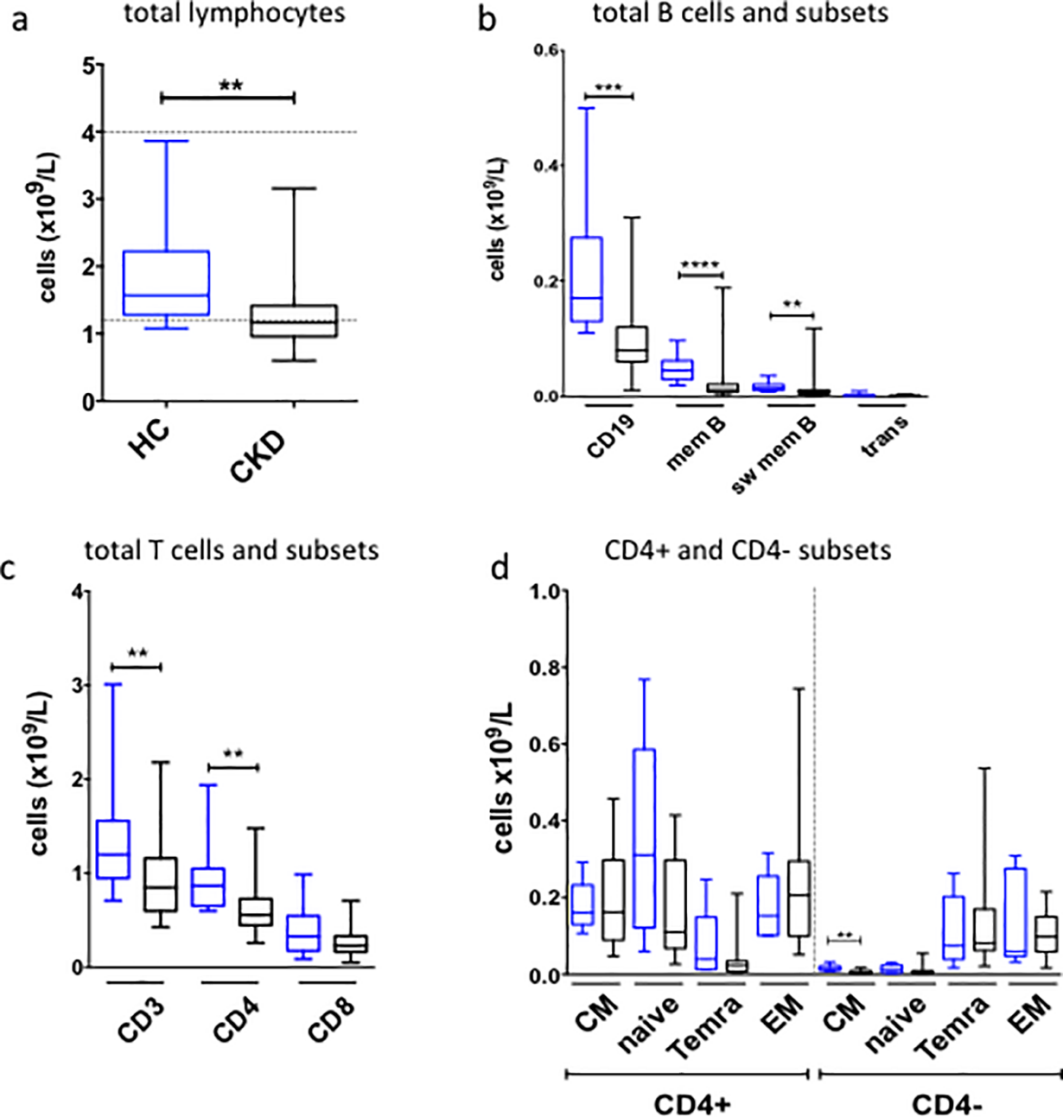
Baseline lymphocyte subsets. **(a-c).** Peripheral blood lymphocyte analysis performed on baseline samples collected from healthy controls (HC; blue) and CKD patients (black) who received hepatitis B vaccination. Absolute counts (HC, n = 12; CKD, n = 25) for total lymphocytes **(a)**, B cell subsets **(b)**, and T cells subsets **(c)**. **(d)**. Memory T cell subsets (HC, n = 6; CKD, n = 16). CM, central memory; EM, effector memory; mem B, memory B cells; sw mem B, switched memory B cells; trans, transitional B cells. All analyses performed using Mann-Whitney U test, ****p<0.0001, ***p<0.001, **p<0.01. Bonferroni correction for α<0.05, p<0.003.

At baseline, patients with CKD were lymphopenic relative to healthy controls (HC) (Fig 2A). Total B cells were significantly reduced in CKD (Fig 2B) due to reductions in memory and switched memory B cells. Median total B cell count in CKD (0.08 x10^9^/L) was below the lower limit of our locally derived normal range (0.14–0.54 x10^9^/L). CKD patients also had significantly lower absolute counts of total and CD4+ T cells (Fig 2C). More detailed analysis of memory T cells revealed a trend towards a reduction in naïve CD4+ T cells (Fig 2D), as reported previously [11]. Both HC and CKD demonstrated median absolute CD8^+^ T cell counts below the lower limit of the diagnostic assay range (0.3–0.9 x10^9^/L), consistent with a decline in this population with age (Fig 2C). Central memory CD4- T cells appeared significantly reduced in CKD, however absolute counts were low in both CKD and HC (Fig 2D). Despite this finding, further analysis demonstrated a correlation between age and total T cells, and age and CD4+ T cells in healthy controls, but not between age and CD8+ T cells (S2 Fig). Age did not correlate with total T cells, CD4+ T cells or CD8+ T cells in CKD patients. There was no correlation between age and total B cells, or age and memory B cells, in either HC or CKD (data not shown). Median values for serum immunoglobulins (Ig) were within normal ranges in both HC (median values–IgG 8.83g/L, IgA 2.13g/L, IgM 0.88g/L) and CKD (median values—IgG 10.14g/L, IgA 2.59g/L, IgM 0.87g/L) (S3 Fig). There was no correlation between age and serum immunoglobulins in either HC or CKD (data not shown).

### Post-vaccination hepatitis B serology

**Fig 3:**
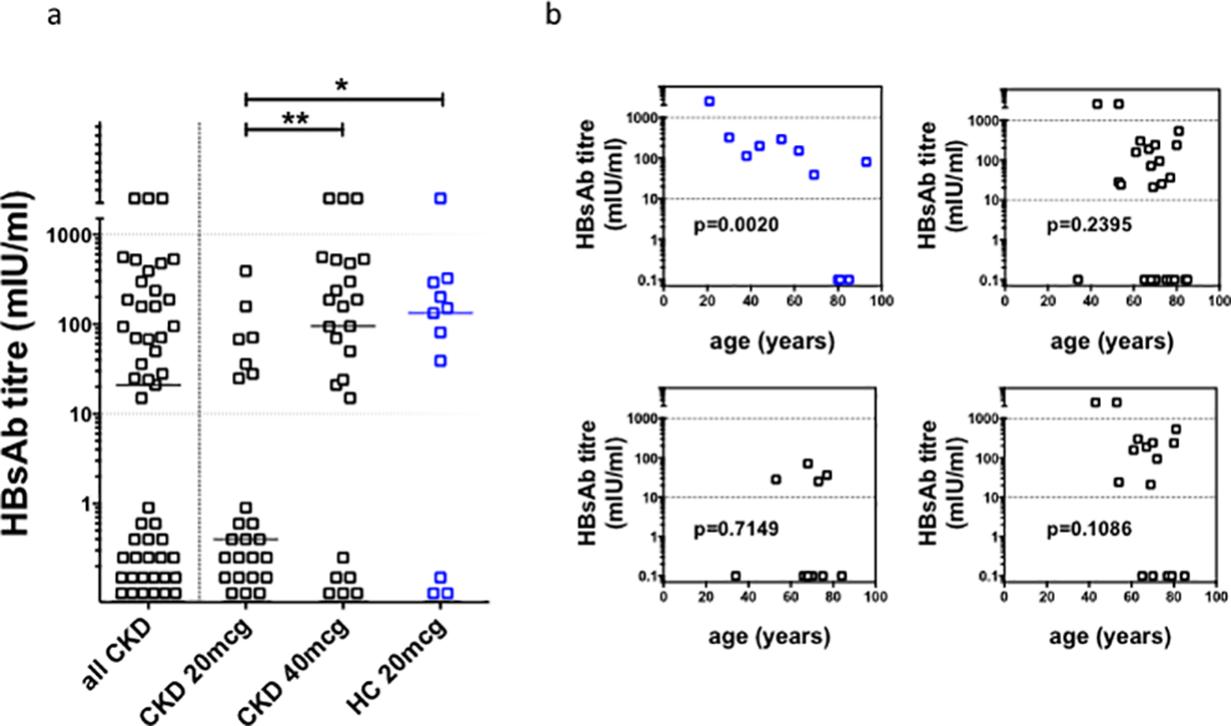
Post–vaccination HBsAb titers. **(a)**. Post-vaccination HBsAb titer for patients (CKD, black symbols; n = 49) and healthy controls (HC; blue symbols, n = 11) who completed primary hepatitis B vaccination schedule. Horizontal dotted line represents working range of serological assay. Horizontal bars indicate medians. Statistical analyses performed using Mann-Whitney U test; *p = 0.0498; **p = 0.0028. Bonferroni correction for α<0.05, p<0.03. **(b).** Rank correlation analysis (Spearman’s test) of age and post vaccination HBsAb titer in healthy controls (n = 11) and CKD patients (n = 49), for both 20mcg CKD group (n = 24) and 40mcg CKD group (n = 25).

As noted in earlier studies, we observed a significant incidence of serological non-responsiveness in CKD (Fig 3A) (47% versus 27% in HC), although CKD responders generated titers of similar magnitude to HC. The median post-vaccination hepatitis B surface antibody (HBsAb) titer was not significantly different between CKD and HC. However, in CKD patients, the 40mcg dose schedule resulted in a significantly higher median post-vaccination HBsAb titer (95mIU/ml vs <10mIU/ml), and reduced the rate of non-responsiveness (24% vs 71%) compared to the 20mcg dose group. Furthermore, only two (8%) of the patients who received the 20mcg schedule attained an HBsAb greater than 100mIU/ml, compared to 12 (28%) of those who received the 40mcg schedule. Hence, overall, the 40mcg schedule in CKD resulted in rates and magnitude of seroconversion comparable to HC. Thus, the serological defect observed in CKD with a 20mcg dose schedule could be overcome by an augmented 40mcg dose schedule.

Age influenced seroconversion independently of CKD. In HC, there was an inverse correlation between age and post-vaccination HBsAb titer (Fig 3B), but this was not observed in CKD, even in the 40mcg schedule group. Despite the negative effect of CKD on seroconversion, we found no direct relationship between eGFR and post-vaccination HBsAb titer (S4 Fig).

#### Longitudinal hepatitis B serology

23 CKD patients and 10 healthy controls, who were recruited to provide both serological and cellular samples between 2011 and 2014, and had provided a 6 week post vaccination sample, were contacted in 2017 to request longitudinal serology (HbsAb). 12 CKD patients and 5 healthy controls provided a repeat serum sample for longitudinal assessment of HBsAb titer. Of the other CKD patients considered for longitudinal serology, 7 were deceased, 2 declined, and two had transferred to other institutions. Of the other healthy controls, 4 were lost to follow up and 1 declined.

Seven of the CKD patients who provided follow-up samples and 4 of the healthy controls were initial serological responders and were included in further analysis. Longitudinal serology for these initial responders is shown in S5 Fig. While the number available for analysis was small, we observed a significant decline in HBsAb titer in CKD; We observed a decline in HBsAb over time in healthy controls (non-significant). The time elapsed between serological measurements was slightly longer for CKD patients (average 1677 days, median 1648 days, range 1330–2150 days) compared to healthy controls (average 1459 days, median 1414 days, range 1052–1956 days).

### Lymphocyte analysis with flow cytometry

#### Baseline cTFH analysis

**Fig 4:**
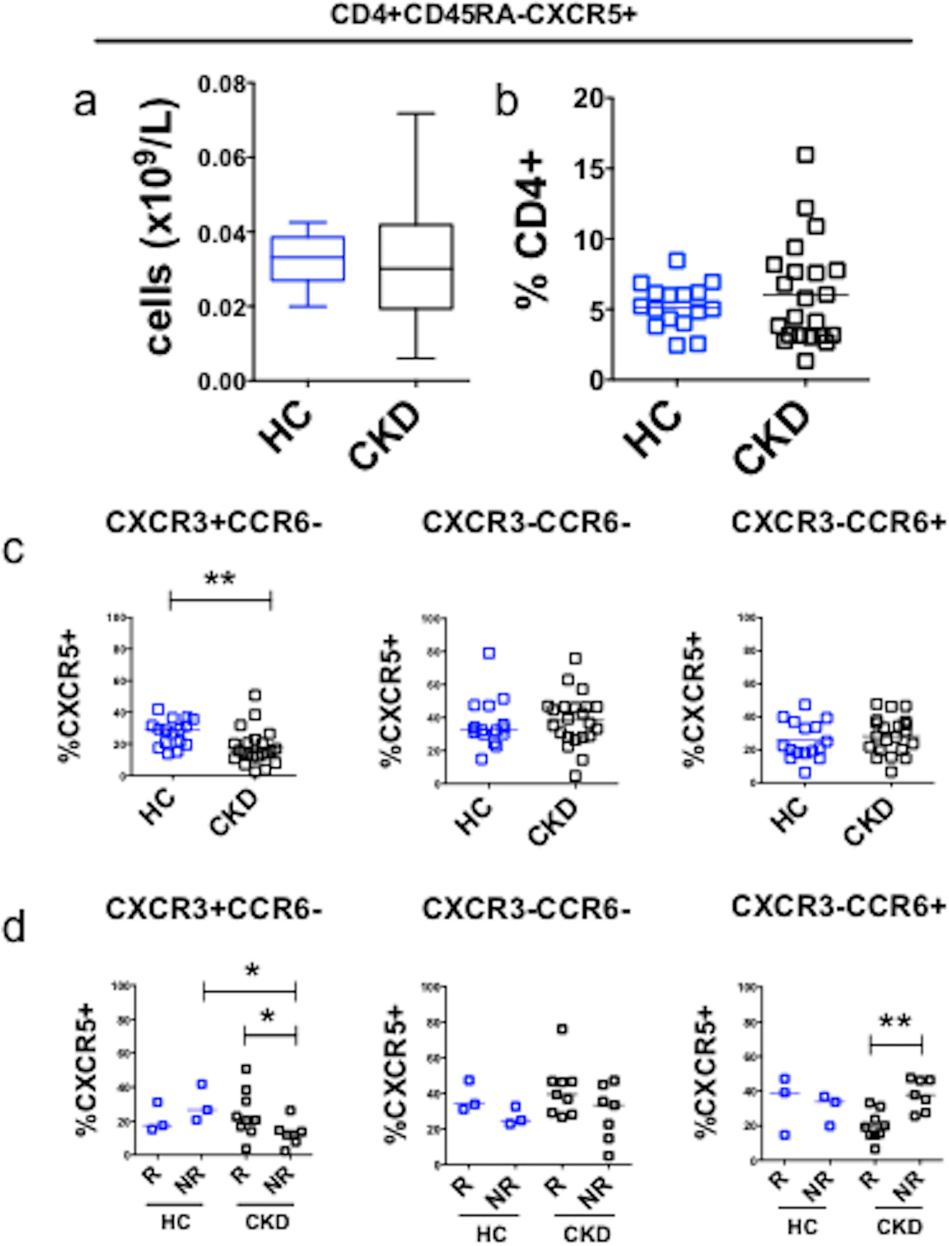
Baseline cTFH subsets in CKD and healthy controls. **(a).** Baseline absolute numbers of CXCR5+memory CD4+ T cells in healthy controls (HC, n = 16, blue) and CKD patients (n = 24, black), including those who subsequently received either HBV or influenza vaccine. **(b).** Baseline proportions of CXCR5+CD45RA- cells expressed as a percentage of CD4+ T cells, in healthy controls (HC, n = 16) and CKD patients (n = 24). **(c).** Baseline cTFH subsets according to CXCR3 and CCR6 surface expression in healthy controls (HC, n = 16) and CKD patients (n = 24). **p<0.01; Bonferroni correction for α<0.05, p<0.016. **(d).** Baseline cTFH subsets analyzed according to subsequent serological response (R, serological responder (HBsAb ≥10mIU/ml); NR, serological non-responder (HBsAb <10mIU/ml)) in healthy controls (HC, n = 6, blue) and CKD patients (n = 19, black) who received HBV. Horizontal bars represent medians. Analysis performed using Mann-Whitney U test: *p<0.05. **p<0.01; Bonferroni correction for α<0.05, p<0.009.

We next examined whether CKD altered baseline proportions of cTFH, and whether this correlated with subsequent serological response to the full hepatitis B vaccination schedule (assay development and gating strategy shown in S6 Fig). Absolute numbers of CXCR5+ memory CD4+ T cells (Fig 4A), and the percentage of CXCR5+CD45RA- cells within the CD4+ T cell compartment were similar between CKD and HC at baseline (Fig 4B). Further analysis showed that CKD patients demonstrated a significant reduction in CXCR3+CCR6- cells as a proportion of CXCR5+ memory CD4+ T cells but not in CXCR3-CCR6- or CXCR3-CCR6+ compartments (Fig 4C). We then analyzed baseline CXCR5+ memory T cell compartments according to subsequent serological response to hepatitis B vaccine. CKD non-responders exhibited lower proportions of CXCR3+CCR6- cTFH at baseline compared to both CKD responders and HC non-responders (Fig 4D).

Next, we analyzed baseline PD-1 expression on cTFH to assess pre-vaccination levels of cellular activation and exhaustion. The abundance of PD-1+ cells within CXCR5+ memory CD4+ T cells, and within each cTFH subset, was similar between HC and CKD (Parts A and B of S7 Fig), and between serological responders and non-responders (Part C of S7 Fig). These findings contrast with previous reports that identified increased expression of activation and apoptosis markers on T cells in CKD [8,31]. Age did not correlate with PD-1+ cTFH or PD-1+ cTFH subsets (data not shown).

#### Defective TFH response in CKD

**Fig 5:**
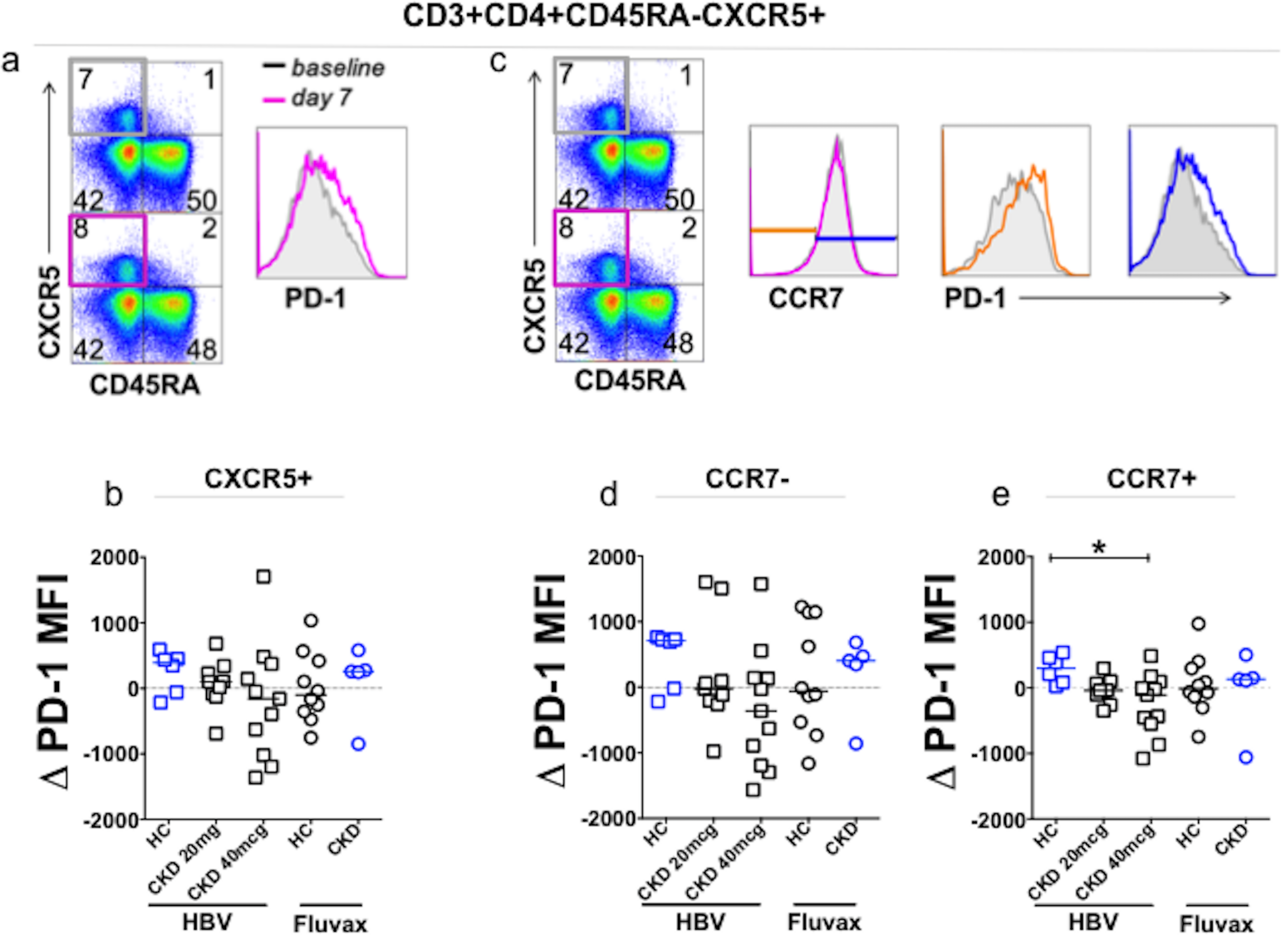
Vaccine induced change in PD-1 expression on CXCR5+ cTFH, and CCR7+ and CCR7- cTFH. **(a)**. Gating strategy to determine PD-1 MFI on CXCR5+ memory CD4+ T cells before and after vaccination. **(b)**. Change in PD-1 MFI on CXCR5+ memory CD4+ T cells after vaccination according to vaccine group. **(c)**. Gating strategy to determine PD-1 MFI according to CCR7 expression in CXCR5+ memory CD4+ T cells. Baseline, grey; Day 7, cerise; CCR7-, d7, orange; CCR7+ d7, blue. **(d-e)**. Vaccine-induced change in PD-1 MFI on **(d)** CCR7- and **(e)** CCR7+ compartments according to vaccine group. CKD, HBV (blue squares, 20mcg n = 9, 40mcg n = 11); HC, HBV (n = 6, blue circles); CKD, Fluvax (blue circles, n = 5); HC, Fluvax (black circles, n = 10). Analysis performed using Mann-Whitney U test, horizontal bars represent medians; *p = 0.0120, Bonferroni correction for α<0.05, p<0.025.

We next compared the T cell compartment before and 7 days after vaccination. cTFH were defined as previously described [28] as CD4+CD45RA-CXCR5+CCR7^lo^ PD-1^hi^ (gating strategy shown in S8 Fig), and expressed as a proportion of memory T cells, or CXCR5^+^ memory T cells. There was no significant change in these parameters after either vaccination, in either HC or CKD (data not shown). There was no correlation between these cTFH parameters and post vaccination HBsAb titers. We analyzed this compartment further by looking for changes in PD-1 mean fluorescence intensity (ΔPD-1 MFI) on CXCR5+ memory T cells (gating strategy Fig 5A). There was no significant change from baseline in either HC or CKD following HBV or Fluvax (Fig 5B).

When ΔPD-1 MFI on CXCR5+ memory cells was further analyzed according to CCR7 expression (gating strategy shown in Fig 5C) we observed a three-fold median increase in PD-1 expression on CCR7+ cells in HC after HBV (Fig 5F), but there was no consistent change in other vaccine groups. This increase was statistically significant when compared to the CKD 40mcg vaccine group, but not the CKD 20mcg vaccine group, or HC or CKD patients who had received Fluvax.

**Fig 6:**
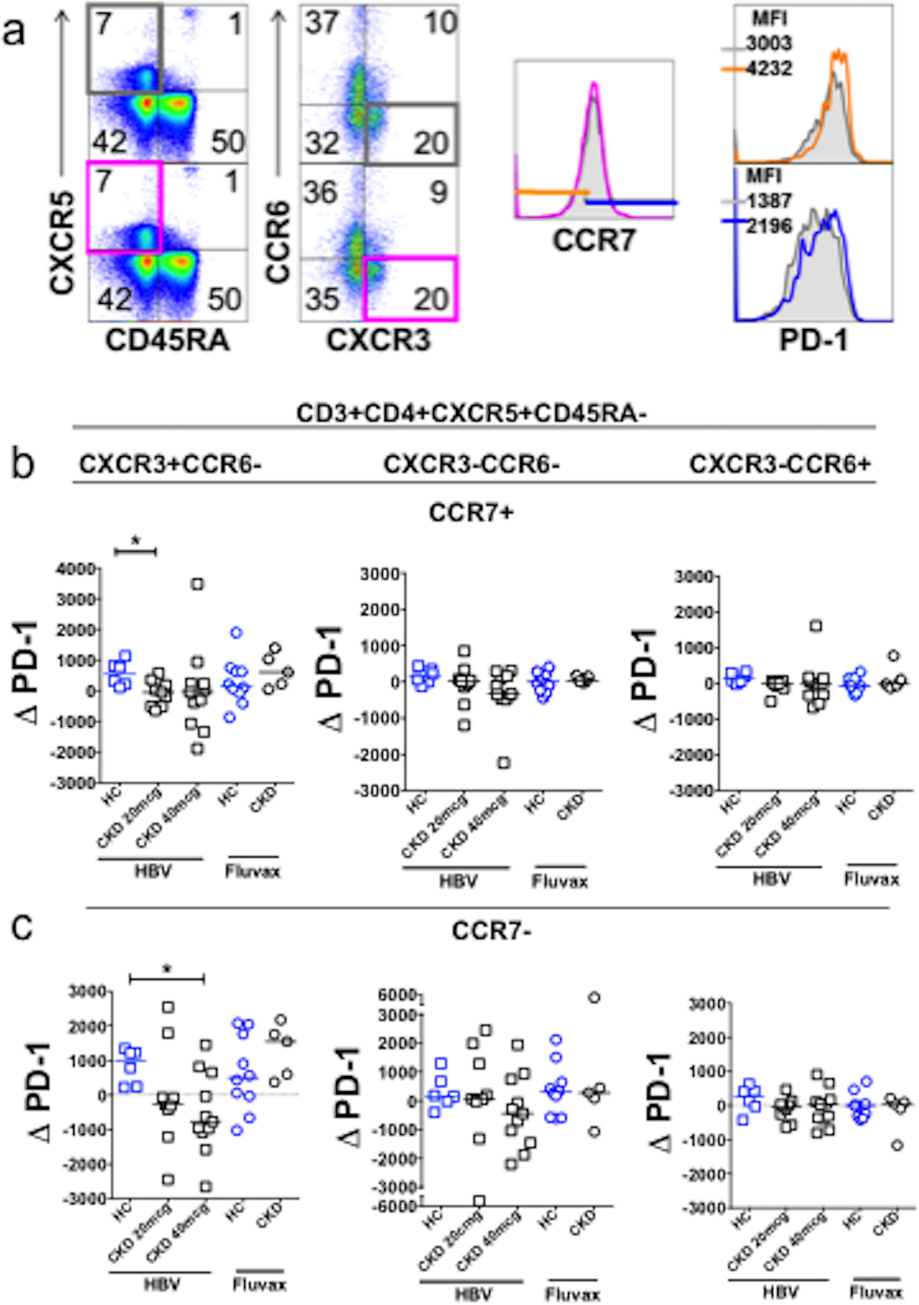
**PD-1 is upregulated on CXCR3+CCR6- cTFH after vaccination, but not after HBV in CKD patients (a)**. Gating strategy to determine PD-1 MFI on CXCR5+ memory CD4+ T cells before (grey box and histograms) and after (colored box and histogram) vaccination, according to CXCR3 and CCR6 expression (CXCR3+CCR6- compartment shown here), followed by CCR7 expression. **(b-c)**. Change in PD-1 MFI in CCR7+ **(b)** and CCR7- **(c)** cTFH compartments after vaccination. HC who received Fluvax (n = 10, blue circles) or HBV (n = 6, blue squares). CKD patients who received HBV (20mcg n = 9, 40mcg n = 11; black squares) or Fluvax (black circles, n = 5). Horizontal bars represent medians. *p<0.05, Bonferroni correction for α<0.05, p<0.025.

cTFH subsets have been demonstrated to undergo dynamic changes after influenza vaccination [23,24]. We therefore examined the abundance and activation of each of these subsets before and after vaccination. In our cohort, neither HBV nor Fluvax elicited changes in the proportions of these subsets (S9 Fig). By contrast, we observed a significant increase in PD-1 expression (as assessed by PD-1 MFI) on both CCR7- and CCR7+ cells within CXCR3+CCR6- cTFH (gating strategy Fig 6A) after HBV in HC, but not in CKD (Fig 6B and 6C). To determine whether failure of PD-1 upregulation on CXCR3+CCR6- cTFH cells was specific to HBV or to CKD, we performed a similar analysis after Fluvax. Interestingly, we observed that seasonal influenza vaccination elicited median increases in PD-1 MFI on both CCR7- and CCR7+ subsets, specifically in CXCR3+CCR6- cTFH, and the magnitude of increase was greater within the CCR7- subset. The responses were similar for magnitude and rate in both CKD and HC.

#### PB response in CKD is equivalent to HC

**Fig 7:**
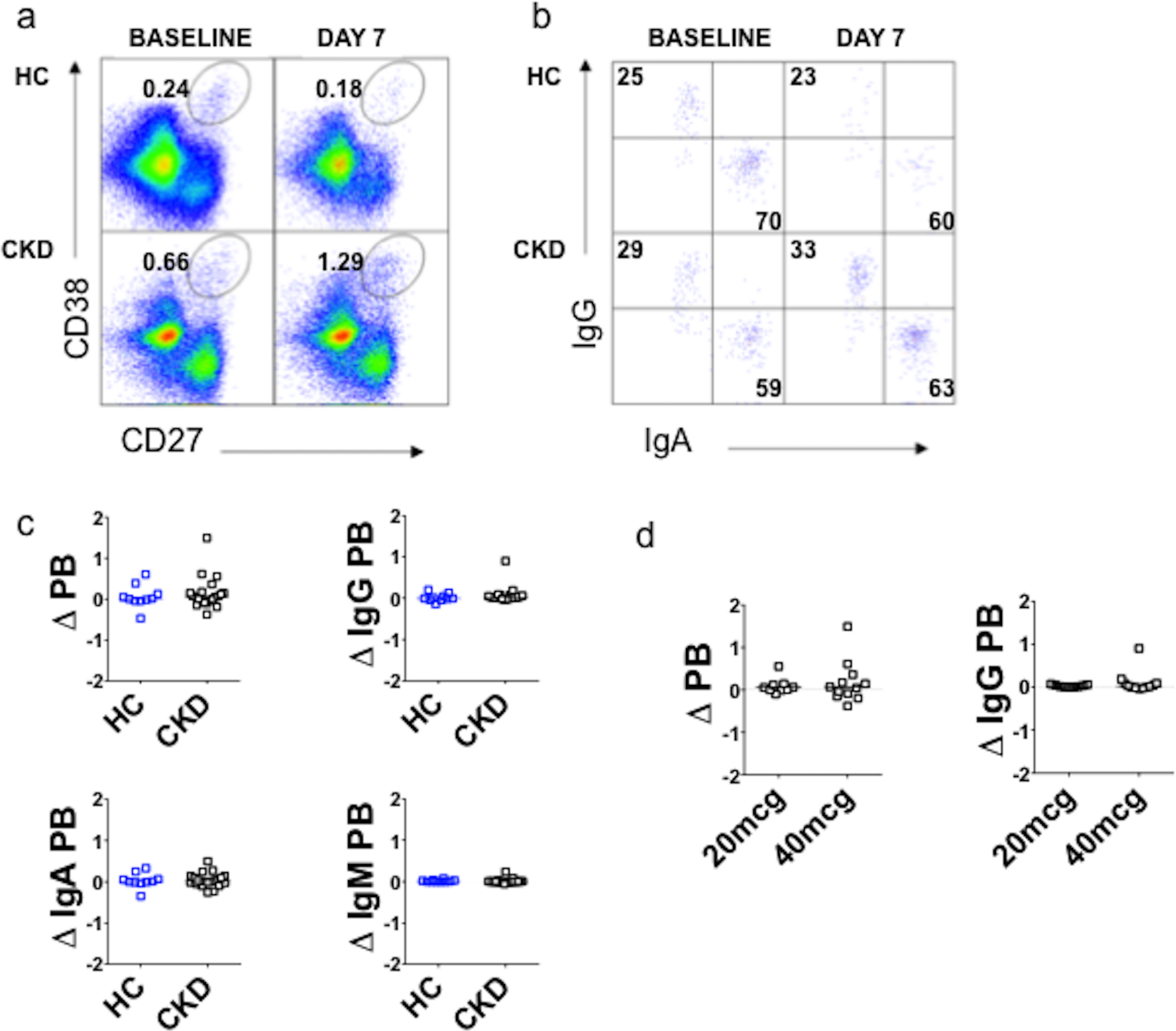
Plasmablasts analysis following HBV. **(a-b)**. Representative FACS analysis for plasmablast enumeration **(a)** and isotyping **(b)** before and 7 days after recombinant hepatitis B surface antigen vaccination. **(c)**. Plasmablast responses for total and isotyped PB. **(d)**. Total and IgG plasmablast responses according to HBV dose schedule (20mcg or 40mcg schedule) in CKD patients. HC, healthy controls (blue), n = 10; CKD, chronic kidney disease patients (black), n = 21; PB, plasmablasts; Δ = change in PB (day 7 minus PB at baseline, when expressed as a percentage of total B cells).

**Fig 8:**
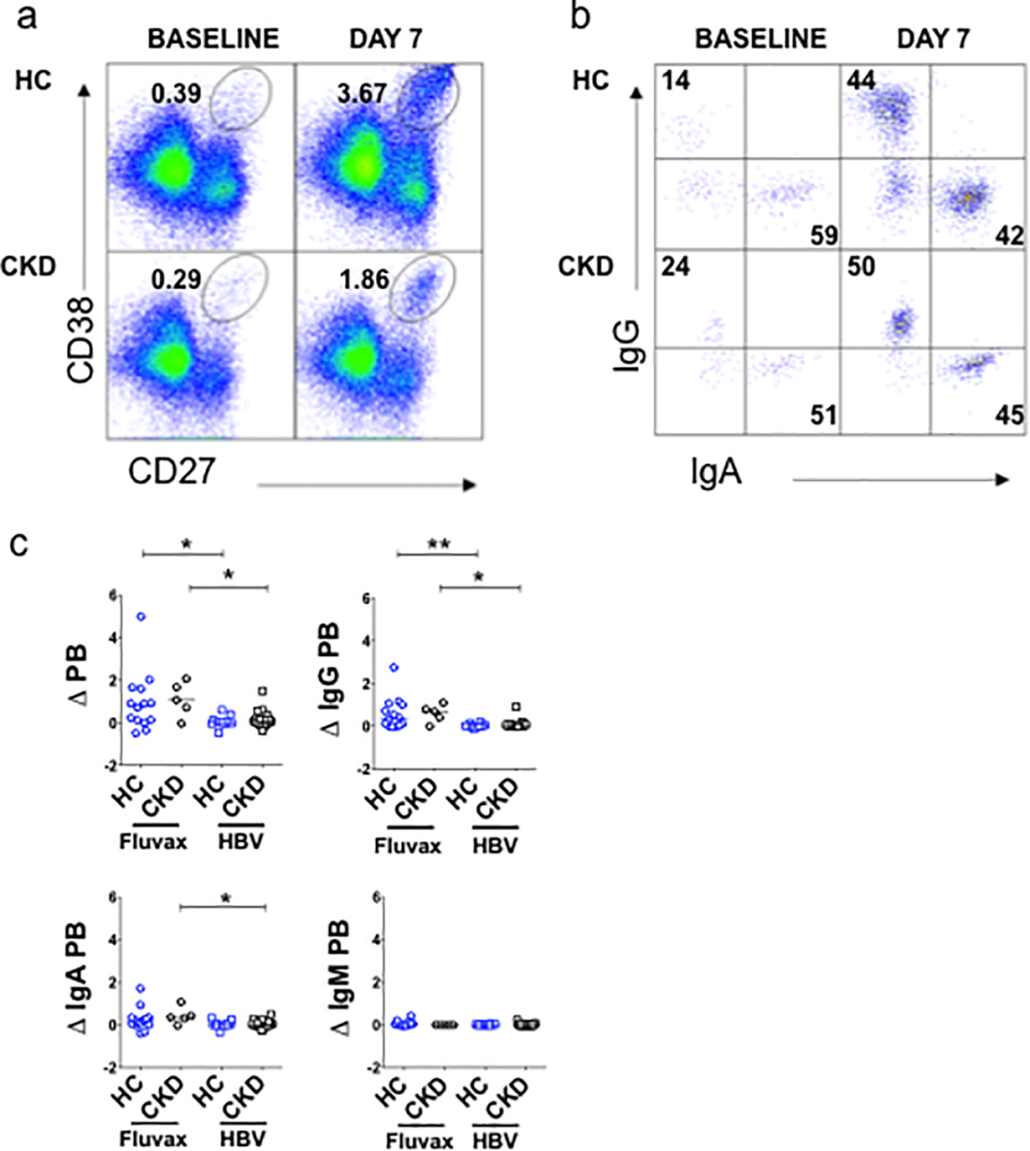
Plasmablast response after seasonal influenza vaccine. **(a-b)**. Representative FACS analysis for plasmablast enumeration **(a)** and isotyping **(b)** before and 7 days after Fluvax. **(c)**. Plasmablast responses for total and isotyped PB, following either Fluvax (circles) or HBV (squares), in either healthy controls (HC, blue) or CKD patients (black). Horizontal bars represent medians. Analyses performed using Mann-Whitney U test. *p<0.05, **p<0.01, Bonferroni correction for α<0.05, p<0.02. HC, healthy controls; CKD, chronic kidney disease; PB, plasmablasts; Δ PB, change in plasmablasts, from baseline to day 7, expressed as a percentage of B cells.

We also examined whether the poor sero-response to HBV in CKD could be explained by differences in PB formation (assay development and gating strategy shown in Parts A and B of S10 Fig). PB were enumerated on day 7 and compared to baseline (expressed as change in proportion of plasmablasts, ΔPB) (Fig 7A). We characterized PB according to isotype, using cytoplasmic staining for IgG, IgA and IgM (Fig 7B). 55% of circulating PB expressed IgA at baseline, whereas 27% expressed IgG (Part C of S10 Fig). We then measured the change in abundance of PB expressing each isotype on day 7 compared to baseline. HBV did not elicit a PB response, nor a change in isotype, within the PB population, in either HC or CKD (Fig 7C), irrespective of HBV vaccine dose (Fig 7D). There was no correlation between PB response and either seroconversion or HBsAb titer after HBV, in either HC or CKD (S11 Fig). Within the CKD cohort, there was no correlation between eGFR and PB response (data not shown). To clarify whether the absence of a PB response was specific to HBV, we compared PB responses to Fluvax in HC and CKD. By contrast, Fluvax elicited a robust PB response of similar magnitude in both HC and CKD (Fig 8A), predominantly accounted for by an increase in IgG PB, with a smaller increase in IgA PB (Fig 8B). The change in total PB, and IgG PB, was significantly greater after Fluvax than after HBV in HC (Fig 6C). A similar difference between vaccination types was seen in CKD, with increases in total and IgA PB significantly greater after Fluvax.

#### PB response correlates with PD-1 upregulation on a subset of cTFH

**Fig 9:**
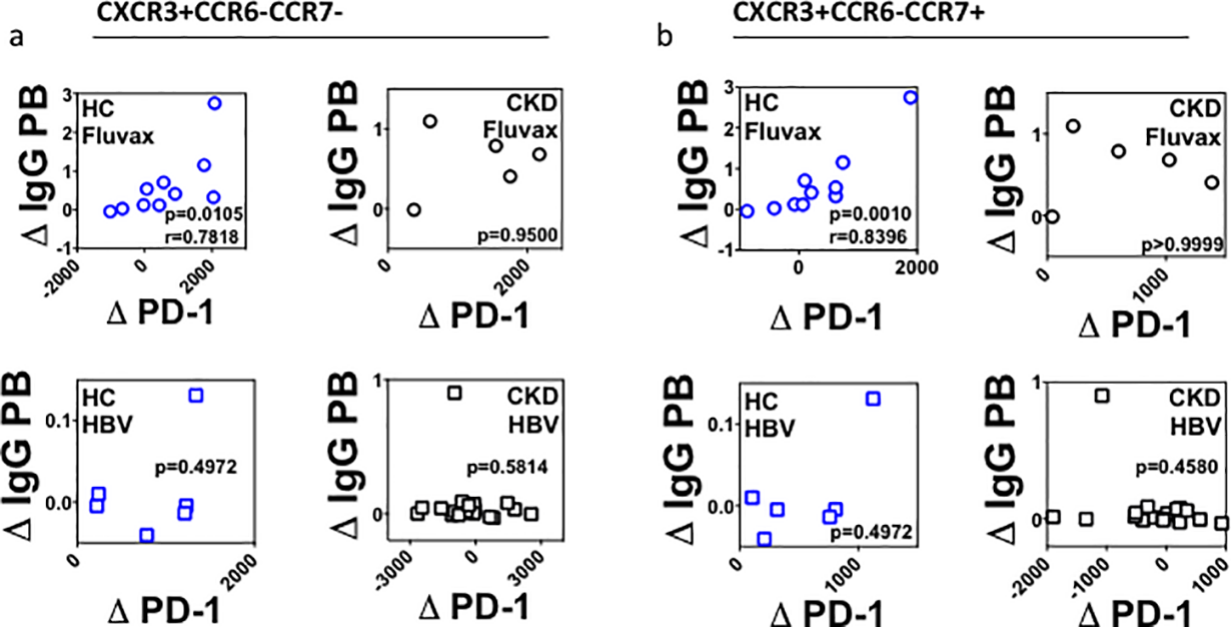
Upregulation of PD-1 on CXCR3+CCR6-CXCR5+ cTFH after Fluvax in healthy controls. **(a-b)**. Relation between IgG PB plasmablast responses and either CCR7- **(a)** or CCR7+ **(b)** CXCR3+ CCR6- CD4+ cTFH cells in healthy controls (HC, blue) or CKD (black) after either Fluvax (circles, upper panels) or HBV (squares, lower panels). Δ IgG PB, change in IgG plasmablasts. Δ PD-1, change in mean fluorescence intensity of PD-1. All analyses performed using Mann-Whitney U test. Correlation analyses performed using Spearman r test.

We have demonstrated that HBV elicits upregulation of PD-1 on CXCR3+ CCR6- cTFH in HC, but not in CKD. By comparison, Fluvax elicited both upregulation of PD-1 on CXCR3+CCR6- cTFH and a PB response, in both HC and CKD. We were therefore interested in whether there might be a correlation between cTFH and PB responses. We found a strong correlation between IgG PB response and PD-1 upregulation within CXCR3+CCR6-CCR7- cTFH and CXCR3+CCR6-CCR7+ cTFH in HC who received Fluvax (Fig 9A and 9B), but not in CKD after Fluvax, or after HBV in any group. Significant correlations were also seen between IgG PB response and CXCR5+ cTFH (Part A of S12 Fig), as well as total CCR7+ and CCR7- cTFH (Parts B and C of S12 Fig). Again no such correlations were seen in CKD, or after HBV in any vaccine group.

## Discussion

End stage renal disease appears to be a state of immune deficiency [1,11,32], with increased risk of infection and of viral infection-related cancer [33]. Detailed information regarding the immune defects conferred by CKD has been difficult to obtain. We characterized memory and effector T and B cell compartments in CKD and examined how these lymphocyte populations respond to vaccination. We observed that a higher proportion of CXCR3+ cTFH at baseline is associated with a more robust response to vaccination, and that activation of this subset of cTFH correlated with vaccine response. At baseline, CKD patients are relatively deficient in the CXCR3+ cTFH subset (Fig 4B), and this is more pronounced in serological non-responders to HBV vaccination (Fig 4C).

Current vaccines depend for their efficacy on generation of high affinity antibodies, which depends in turn on affinity maturation within GC reactions, wherein proliferating B cells acquire somatic mutations in Ig genes, and are selected according to antigen affinity [17, 34–37]. Survival depends on provision of T cell help. TFH cells were first described as follicle-resident T cells, and exhibit a unique capacity to provide B cell help, including within GC [18, 38, 39]. GC are relatively inaccessible in humans, but a subset of circulating memory T cells shares characteristics with GC TFH, including the capacity to provide B cells with help [24,25,28,39,40]. We observed an increase in PD-1 expression on the CXCR3+ subset of cTFH after HBV vaccination of healthy controls, but not in CKD patients (Fig 6). Upregulation of PD-1 does not reflect increased abundance of PD-1+ cTFH cells after vaccination, but suggests activation rather than proliferation or increased release of this subset from GC into the circulation.

Interestingly, we observed similar numbers of CXCR5+ CD4+ T cells in CKD and age-matched controls, and identified a deficiency in the CXCR3+ subset of CXCR5+ cTFH cells in CKD. Our study population was predominantly elderly. Another recent study identified a relative deficiency of cTFH in elderly individuals (without CKD) [41], and also reported that cTFH cells in the elderly express higher levels of ICOS, consistent with a higher background level of T cell activation. ICOS expression correlates with PD-1 expression on cTFH in both health and autoimmune disease [26]. We examined PD-1 as a marker of baseline T cell activation, however, did not observe any correlation between age and expression of PD-1+ on cTFH. Abundance of PD-1+ CXCR5+ cells has been shown to correlate with influenza vaccine responsiveness in the young but not in the elderly [41], which could account for why baseline PD-1 expression did not distinguish sero-responders, or between CKD and HC, in our mostly elderly cohort.

PD-1 is upregulated after stimulation through the T cell receptor, and is a negative regulator of T cell activation and proliferation [42], including on GC TFH where PD-1 is expressed at high levels [43]. In HC, we observed that serological response to both Fluvax and HBV correlates with induction of PD-1 expression by CXCR3+ cTFH. The defect in induction in CKD is not absolute, since it was observed in CKD patients after Fluvax. We observed a correlation between induction of PD-1 on CXCR3+ cTFH and PB induction in health controls after Fluvax. This response was observed in both CCR7+ and CCR7- subsets of cTFH, and although the response was of greater magnitude in the CCR7- compartment, induction of PD-1 on CCR7+ cells correlated best with IgG PB response. These findings are consistent with induction the of PD-1 on cTFH as a readout of a productive T-dependent B cell response. By contrast, the absence of a corresponding PB response after HBV indicates an origin of cTFH activation other than germinal center(s), such as via activation of mature naïve T cells in a B cell-independent fashion [18]. The defect in CKD would therefore appear to be within B-independent T cell responses.

Previous studies suggested that CCR6+ cTFH correlate with established or chronic antibody responses. In juvenile dermatomyositis, an increased proportion of CXCR3-CCR6+ cTFH has been reported to correlate with both disease activity and circulating PB numbers [24]. Likewise, abundance of PD-1^low^ CXCR3- cTFH correlates with the presence of highly specific, broadly neutralizing antibodies against HIV [25]. By comparison, data reported here is consistent with other results that show that the CXCR3+ cTFH subset responds to immediate antigenic challenge, particularly after influenza vaccination [23]. The absence of expansion of CXCR3+ cTFH in our study might reflect a difference between the elderly and children, since we did not observe expansion of the CXCR3+ subset in HC either.

One possible model to account for our findings and those reported previously is that different cTFH subsets act at different stages in the generation of specific antibody responses. Activation of CXCR3+ CCR6- cTFH is observed in response to both novel and recall antigenic challenges, as suggested by their activation after both influenza vaccine and HBV. Conversely, CXCR3- (predominantly CCR6+) cTFH correlate with the magnitude and maintenance of the subsequent antibody response, perhaps in response to repeated or chronic antigenic stimulus, as evidenced by their correlation with specific antibody responses in chronic autoimmune disease and HIV. The integrity of serological immunity function depends on both baseline proportions of cTFH subsets and their activation state, as suggested by the apparent inability of hyper-activated, ICOS+ cTFH to support specific antibody responses in the elderly, and dysregulation of cTFH in autoimmune disease.

The results of this study are limited by several factors. First, the CKD patients were not blinded to their group allocation. In theory, this may have affected compliance with completion of the full vaccination schedule, but there was no difference in discontinuation rates between the vaccine dose groups. There was no 40mcg dose control group, which may have been informative in further clarifying serological and cellular defects seen in CKD. The risk of type II error may have been increased by failure to enrol the pre-determined number of patients (81 CKD patients), particularly for several cellular correlates of a serological defects in CKD, nevertheless, we demonstrated a significant serological response to the augmented vaccine dose in CKD patients.

In summary, we have demonstrated for the first time that a four-dose schedule of 40mcg of HBV is superior to a 20mcg four-dose schedule when administered to patients with CKD prior to initiation of dialysis. The augmented schedule resulted in superior rate and magnitude of seroconversion. Only the 40mcg schedule elicited HBsAb titers greater than 100mIU/ml, a preferential cutoff in CKD patients commencing dialysis, as it is associated with longer duration of sero-protection [5]. These results suggest that a four-dose regimen of 40mcg should be administered routinely to all CKD patients when in preparation for dialysis. Recently, HBV using a recombinant antigen conjugated with a TLR9-agonist exhibited improved seroconversion in CKD [44]. While the cellular mechanism to account for this response remains to be determined, one hypothesis is activation of Th-1 CD4+ T cells [44]. The findings reported here provide an opportunity to test the cellular basis of this finding, in order to optimize vaccine responses in patients with CKD.

## Supporting information

S1 Fig Summary of enrolment and exclusion. Flow diagram of enrolment and sample collection for healthy controls and CKD patients who received hepatitis B vaccine. All healthy controls received a standard dose of hepatitis B vaccine of 20mcg, and were recruited for both cellular and serological sample collection. *on Methotrexate; CKD—chronic kidney disease; HBsAb–hepatitis B surface antibody; PB–plasmablast; TFH–follicular helper T cell.
(TIF)

S2 Fig Age related changes in lymphocytes. Baseline total T cells, CD4+ T cells and CD8+ T cells, compared to age, in healthy controls and CKD patients who subsequently received hepatitis B vaccination. HC, healthy controls (blue squares, n = 10), CKD, chronic kidney disease (black squares, n = 27). Correlation analysis performed using Spearman test.
(TIF)

S3 Fig Baseline serum immunoglobulin. Immunoglobulin measurements in healthy controls (HC, blue, n = 10) and CKD patients (black, n = 28) who subsequently received hepatitis B vaccination. Dashed horizontal lines represent working range of serological assay. Horizontal bars are medians, statistical analysis performed using Mann-‐Whitney U test.
(TIF)

S4 Fig Post-vaccination HBsAb titer compared by eGFR. Comparison of vaccine responses and baseline eGFR in (**a**) all CKD patients, and CKD patients who received the (**b**) 20mcg or (**c**) 40mcg vaccine schedule. Horizontal dashed lines represent upper and lower limits of HBsAb assay detection. Statistical analyses performed using Spearman test. HBsAb, hepatitis B surface antibody; eGFR, estimated glomerular filtration rate.
(TIF)

S5 Fig Longitudinal HBV serology. HBV serology for (**a**) healthy controls and (**b**) CKD patients, who initially responded to HBV. Change in titer was significant for CKD patients using the Wilcoxon signed rank test. Statistical significance was not reached for titer decline in healthy controls, but a clear trend was also evident. Time between initial serology (a) taken six weeks after primary Hepatitis B vaccination course, and longitudinal serology (b) was longer for healthy controls (average 1677 days, median 1648 days) than CKD patients (average 1459 days, median 1414 days). HBsAb–hepatitis B surface antibody; CKD–chronic kidney disease.
(TIF)

S6 Fig TFH gating strategy. **(a)**. Staining and gating strategy for Tfh cells was performed using human tonsil. After gating on each of the four populations within the CXCR5 CD45RA plot, cells were analyzed for CXCR3 versus CCR6 expression, and CCR7 versus PD-1 expression to identify the population of interest. CXCR5hi PD-1hi bona fide TFH cells are rare in peripheral blood (red square). The cells most closely resembling circulating counterparts of TFH in tonsil are CXCR5+PD-1+ cells (blue square), and therefore peripheral blood staining was based on this phenotype. **(b)**. Gating strategy to determine CXCR5+ memory cells, subsets based on CXCR3 and CCR6 expression, and PD-1 expression. This was compared to CXCR3, CCR6 and PD-1 expression on CXCR5-CD45RA+ naive CD4+ T cells. After gating on CD4+ T cells, CXCR5+ memory cells (pink) and naive cells (orange) were then analyzed for CXCR3 and CCR6 expression. Most naive CD4+ T cells are CXCR3-CCR6- CCR7+, and do not express PD-1. Therefore, CXCR3-CCR6- naive cells were used to determine the PD-1 and CCR7 gates on CXCR5+ memory cell subsets, including for abundance of PD-1+ cells (histogram) irrespective of CCR7 expression. All values shown are percentages.
(TIF)

S7 Fig Baseline expression of PD-1 on cTFH and cTFH subsets. (a). Baseline PD-1+ cells as a percentage of CXCR5+ memory CD4+ T cells. (b). Baseline PD-1+ cells within each cTFH subset, and expressed as a percentage of that subset, in all healthy controls (blue squares, n = 16) and all CKD patients (black squares, n = 22). (c). Baseline PD-1+ cells in cTFH subsets, displayed according to subsequent sero-responsiveness, in healthy controls (HC, blue squares) and CKD patients (CKD, black squares), who subsequently received hepatitis B vaccine and had post vaccination HBsAb measurement performed. Horizontal bars represent medians; analysis performed using Mann-Whitney U test. R–sero-responder (HBsAb ≥10mIU/ml); NR–sero-non-responder (HBsAb <10mIU/ml).
(TIF)

S8 Fig Identification of cTFH in peripheral blood. Gating strategy for circulating TFH. After gating on forward and side scatter for lymphocytes, CD3+CD4+CD45RA-CXCR5+ cells were analyzed for expression of PD-1 and CCR7, on samples collected at baseline (before vaccination), and 7 days after vaccination. All values are percentages.
(TIF)

S9 Fig TFH after vaccination. Change (Δ) in TFH subsets (cells as a % of CXCR5+ cells) after vaccination, according to vaccine type and clinical group. HC–healthy controls; CKD–chronic kidney disease; Fluvax–seasonal influenza vaccination; HBV–hepatitis B vaccine. Horizontal bars represent medians.
(TIF)

S10 Fig Plasmablast identification and analysis. (a) Flow cytometric analysis of human tonsil for the identification of plasmablasts. After gating on lymphocytes according to forward and side scatter, cell populations are selected which are negative for CD3 and CD14 expression, positive for CD19, CD27 and CD38 (oval gate), and express high levels of MHC class II. (b). The same staining and gating strategy is applied to the analysis of peripheral blood. Since plasmablasts downregulate CD19, this gate is set to capture CD19lo cells. Analysis before (*top row*) and seven days after (*bottom row*) seasonal influenza vaccination in a healthy control identifies an increase in plasmablasts at day 7, which predominantly express cytoplasmic IgG. (c). Baseline (prevaccination) proportions of plasmablasts according to cytoplasmic immunoglobulin (Ig) isotype expression in healthy controls (HC-blue circles) and CKD patients (black circles); each circles represents an individual. Horizontal bars are medians. PB–plasmablasts.
(TIF)

S11 Fig Relationship between plasmablast response and post-vaccination HBsAb titer. Healthy controls (HC, blue squares) and CKD patients (CKD, black squares); Δ PB—change in total plasmablasts expressed as a percentage of total B cells; (Δ IgG PB) change in IgG plasmablasts expressed as a percentage of total B cells. Dashed horizontal lines represent upper and lower limits of detection of HBsAb assay. HBsAb–hepatitis B surface antibody. Statistical analyses performed using Spearman test of correlation.
(TIF)

S12 Fig Correlation between the vaccine–induced change in circulating IgG plasmablasts. Examination of the relation between change in plasmabalstst (Δ IgG PB) and the change in PD-1 MFI (ΔPD-1) on (**a**) cTFH, (**b**) CCR7+ cTFH and (**c**) CCR7- TFH subsets, according to vaccine group. HC–healthy controls; CKD–chronic kidney disease; Fluvax—seasonal influenza vaccine; HBV—hepatitis B vaccine; HBsAb, -hepatitis B surface antibody. Correlation analysis performed using Spearman test.
(TIF)

S1 File HREC original submission 2011. (PDF)

S2 File CONSORT 2010 checklist E da Silva. (PDF)
