# Supplementary figures and images for: A randomized trial of serological and cellular responses to hepatitis B vaccination in chronic kidney disease
Source: PLoS One. 2018 Oct 10;13(10):e0204477. doi: 10.1371/journal.pone.0204477 (PMC6179249; doi:10.1371/journal.pone.0204477)

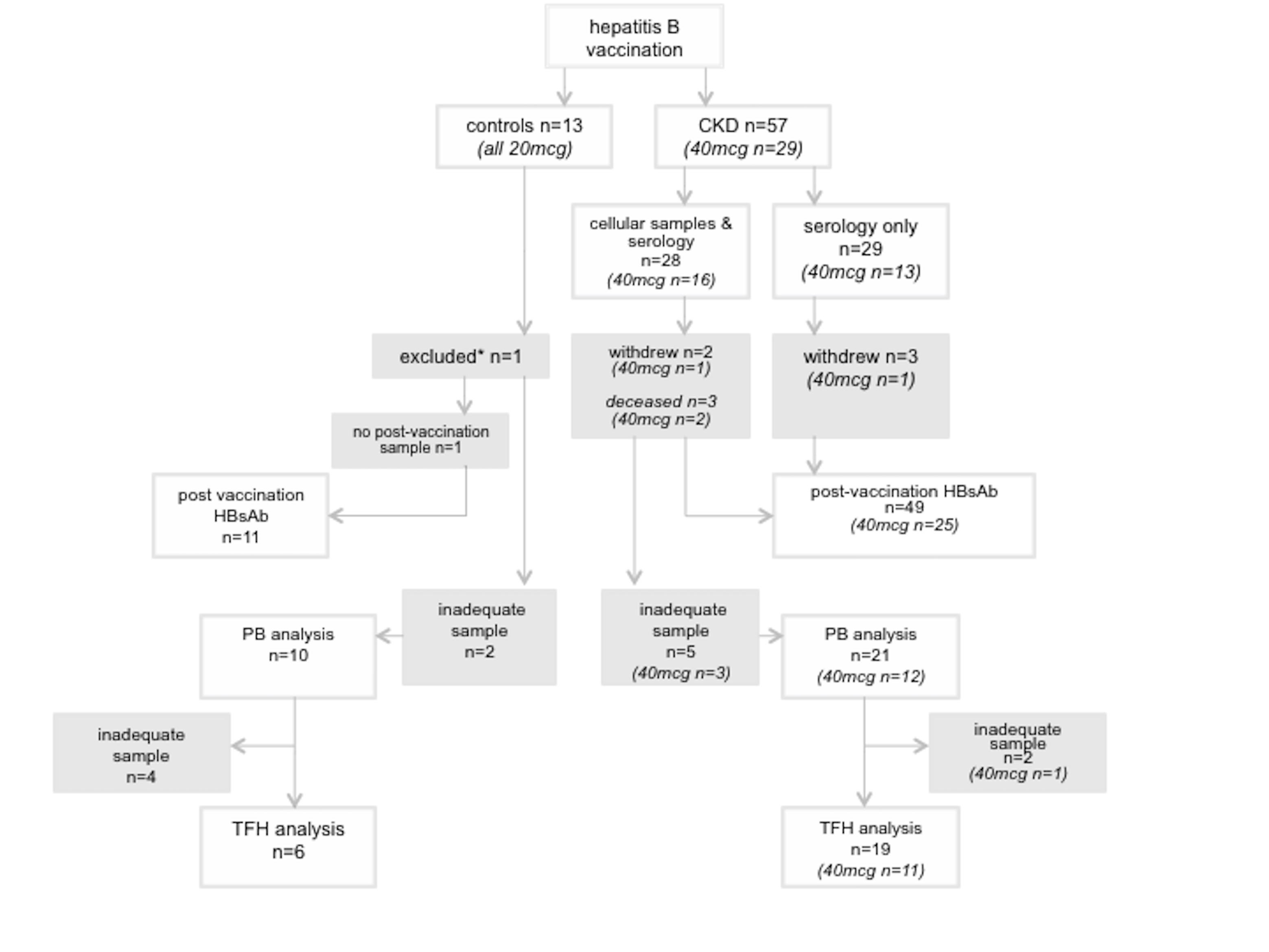

Supplement: S1 Fig — Flow diagram of enrolment and sample collection for healthy controls and CKD patients who received hepatitis B vaccine. All healthy controls received a standard dose of hepatitis B vaccine of 20mcg, and were recruited for both cellular and serological sample collection. *on Methotrexate; CKD—chronic kidney disease; HBsAb–hepatitis B surface antibody; PB–plasmablast; TFH–follicular helper T cell. (TIF) [file pone.0204477.s001.tif]

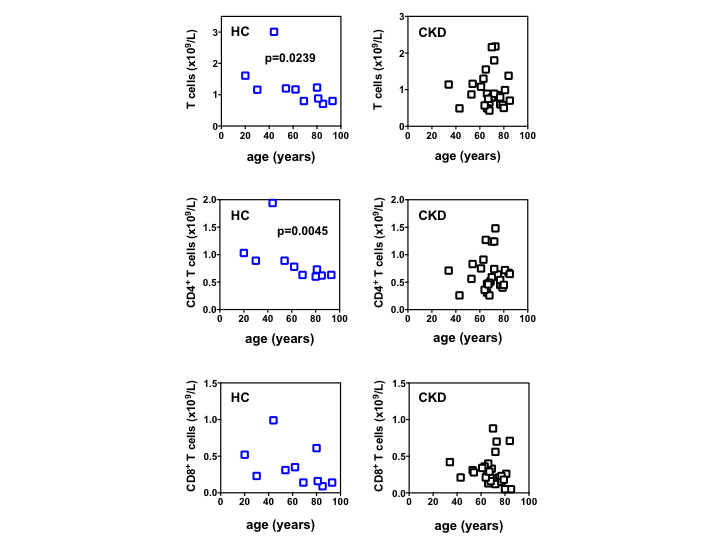

Supplement: S2 Fig — Baseline total T cells, CD4+ T cells and CD8+ T cells, compared to age, in healthy controls and CKD patients who subsequently received hepatitis B vaccination. HC, healthy controls (blue squares, n = 10), CKD, chronic kidney disease (black squares, n = 27). Correlation analysis performed using Spearman test. (TIF) [file pone.0204477.s002.tif]

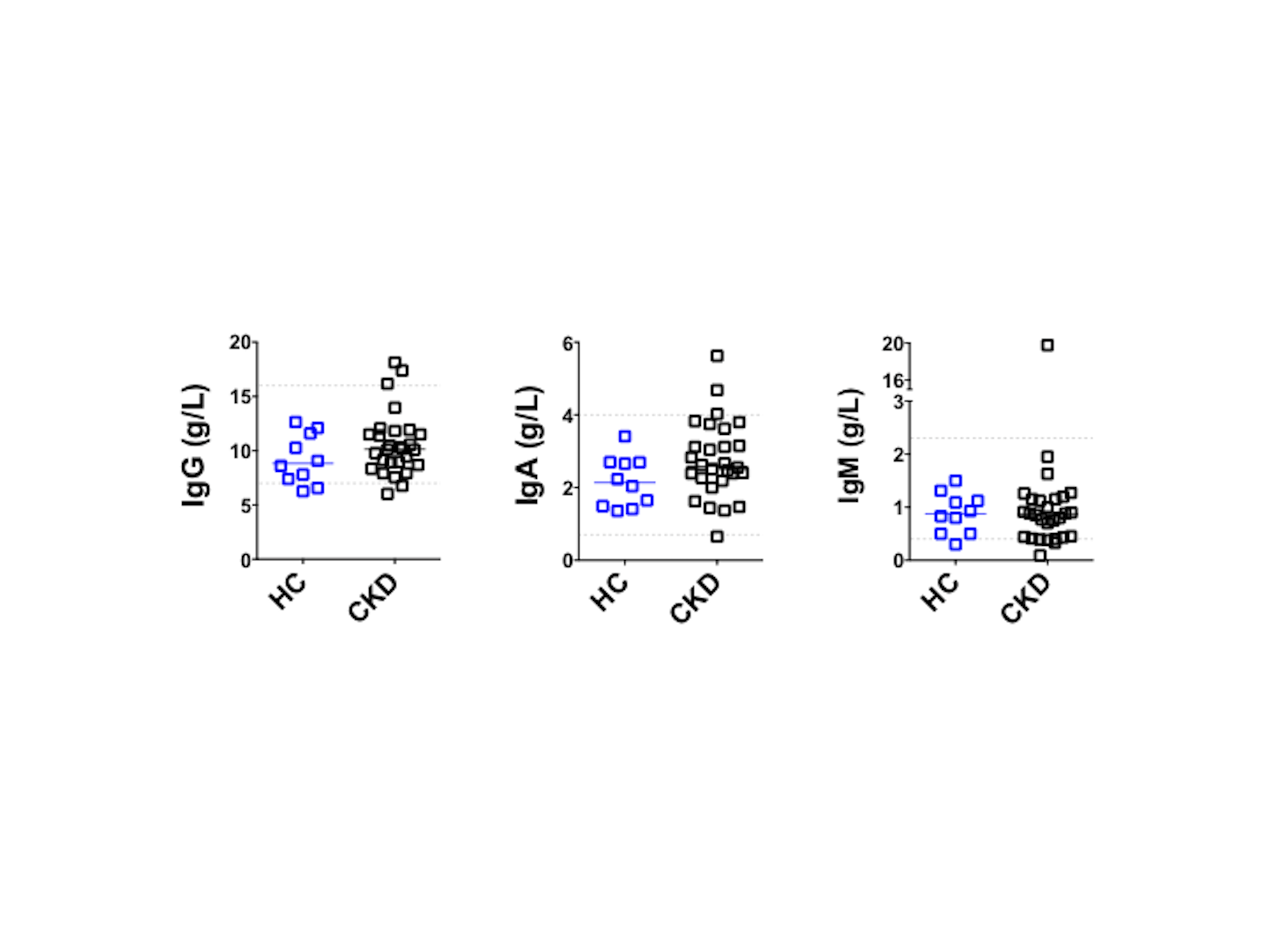

Supplement: S3 Fig — Immunoglobulin measurements in healthy controls (HC, blue, n = 10) and CKD patients (black, n = 28) who subsequently received hepatitis B vaccination. Dashed horizontal lines represent working range of serological assay. Horizontal bars are medians, statistical analysis performed using Mann-‐Whitney U test. (TIF) [file pone.0204477.s003.tif]

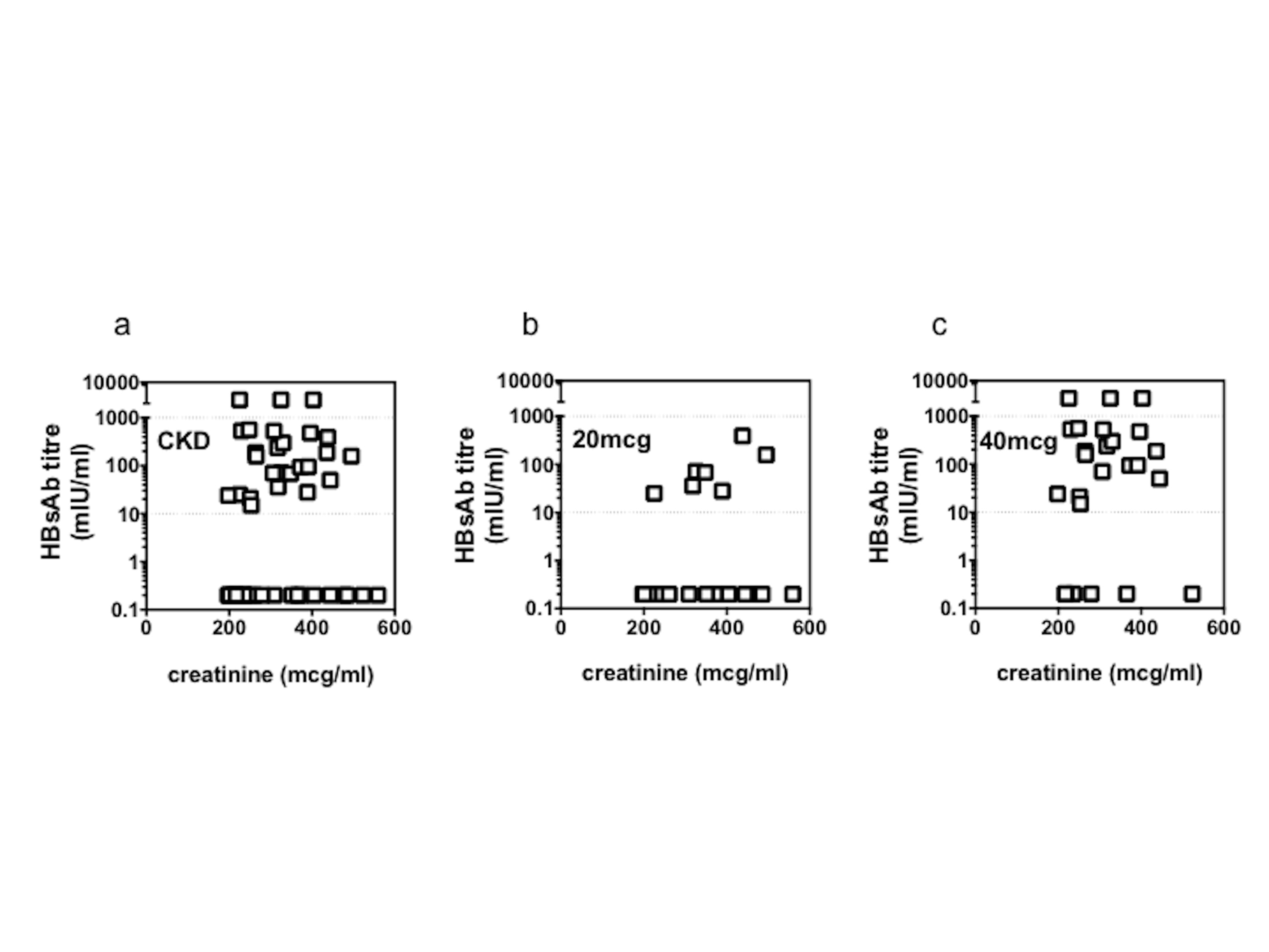

Supplement: S4 Fig — Comparison of vaccine responses and baseline eGFR in (a) all CKD patients, and CKD patients who received the (b) 20mcg or (c) 40mcg vaccine schedule. Horizontal dashed lines represent upper and lower limits of HBsAb assay detection. Statistical analyses performed using Spearman test. HBsAb, hepatitis B surface antibody; eGFR, estimated glomerular filtration rate. (TIF) [file pone.0204477.s004.tif]

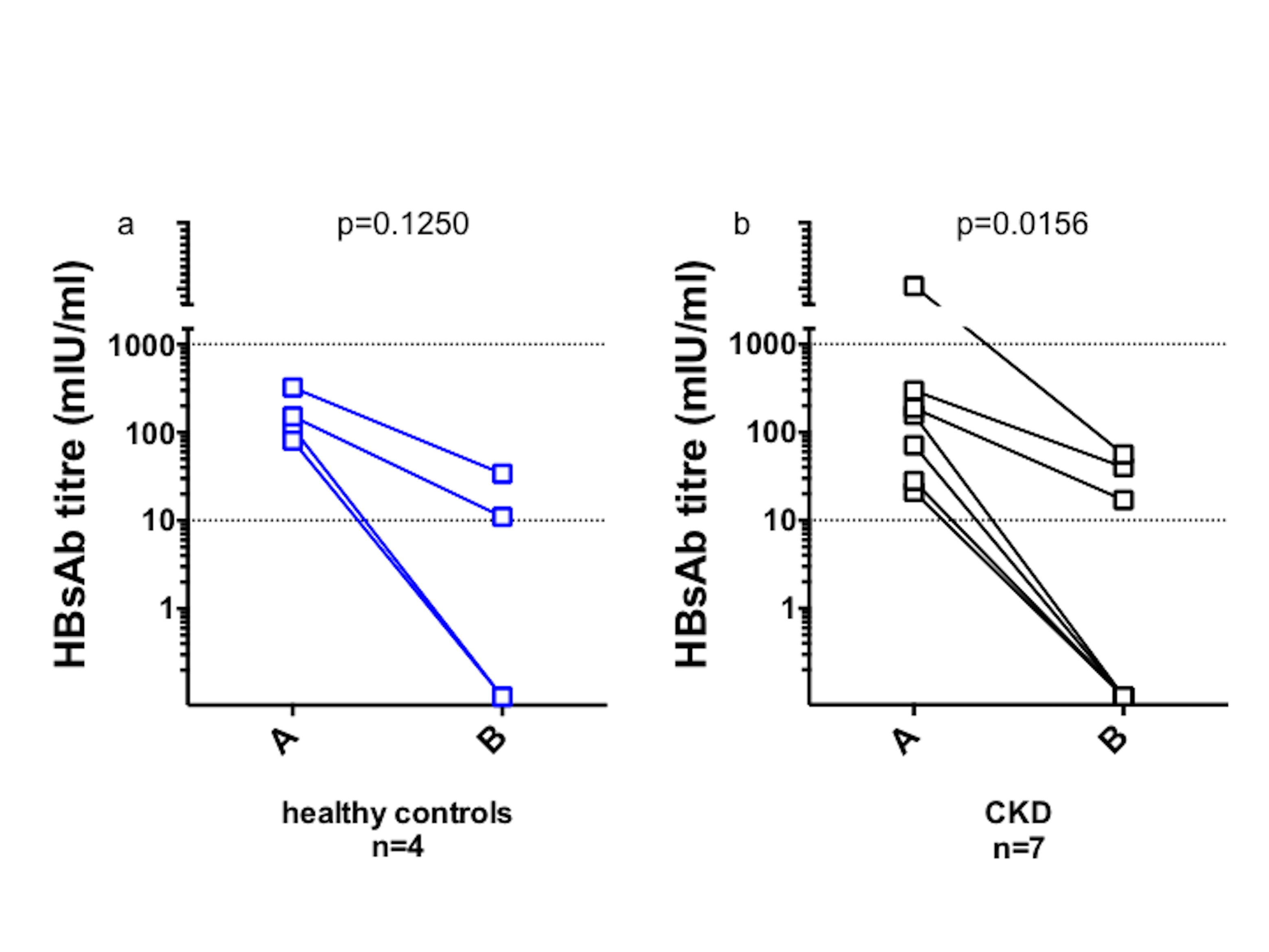

Supplement: S5 Fig — HBV serology for (a) healthy controls and (b) CKD patients, who initially responded to HBV. Change in titer was significant for CKD patients using the Wilcoxon signed rank test. Statistical significance was not reached for titer decline in healthy controls, but a clear trend was also evident. Time between initial serology (a) taken six weeks after primary Hepatitis B vaccination course, and longitudinal serology (b) was longer for healthy controls (average 1677 days, median 1648 days) than CKD patients (average 1459 days, median 1414 days). HBsAb–hepatitis B surface antibody; CKD–chronic kidney disease. (TIF) [file pone.0204477.s005.tif]

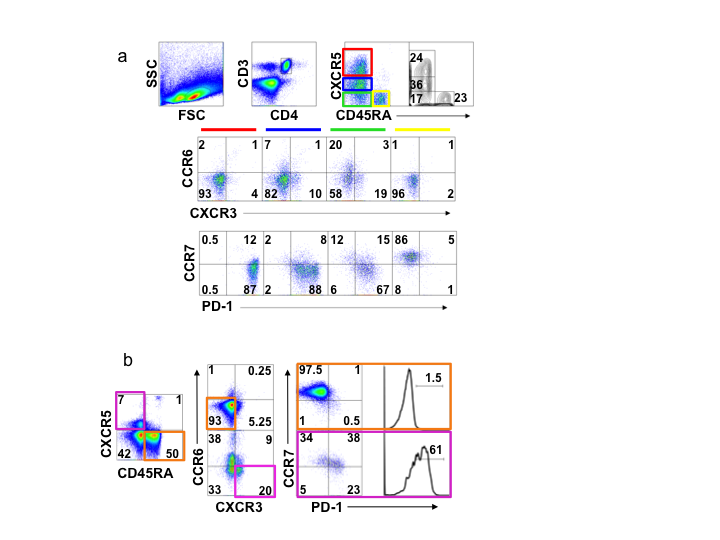

Supplement: S6 Fig — (a). Staining and gating strategy for Tfh cells was performed using human tonsil. After gating on each of the four populations within the CXCR5 CD45RA plot, cells were analyzed for CXCR3 versus CCR6 expression, and CCR7 versus PD-1 expression to identify the population of interest. CXCR5hi PD-1hi bona fide TFH cells are rare in peripheral blood (red square). The cells most closely resembling circulating counterparts of TFH in tonsil are CXCR5+PD-1+ cells (blue square), and therefore peripheral blood staining was based on this phenotype. (b). Gating strategy to determine CXCR5+ memory cells, subsets based on CXCR3 and CCR6 expression, and PD-1 expression. This was compared to CXCR3, CCR6 and PD-1 expression on CXCR5-CD45RA+ naive CD4+ T cells. After gating on CD4+ T cells, CXCR5+ memory cells (pink) and naive cells (orange) were then analyzed for CXCR3 and CCR6 expression. Most naive CD4+ T cells are CXCR3-CCR6- CCR7+, and do not express PD-1. Therefore, CXCR3-CCR6- naive cells were used to determine the PD-1 and CCR7 gates on CXCR5+ memory cell subsets, including for abundance of PD-1+ cells (histogram) irrespective of CCR7 expression. All values shown are percentages. (TIF) [file pone.0204477.s006.tif]

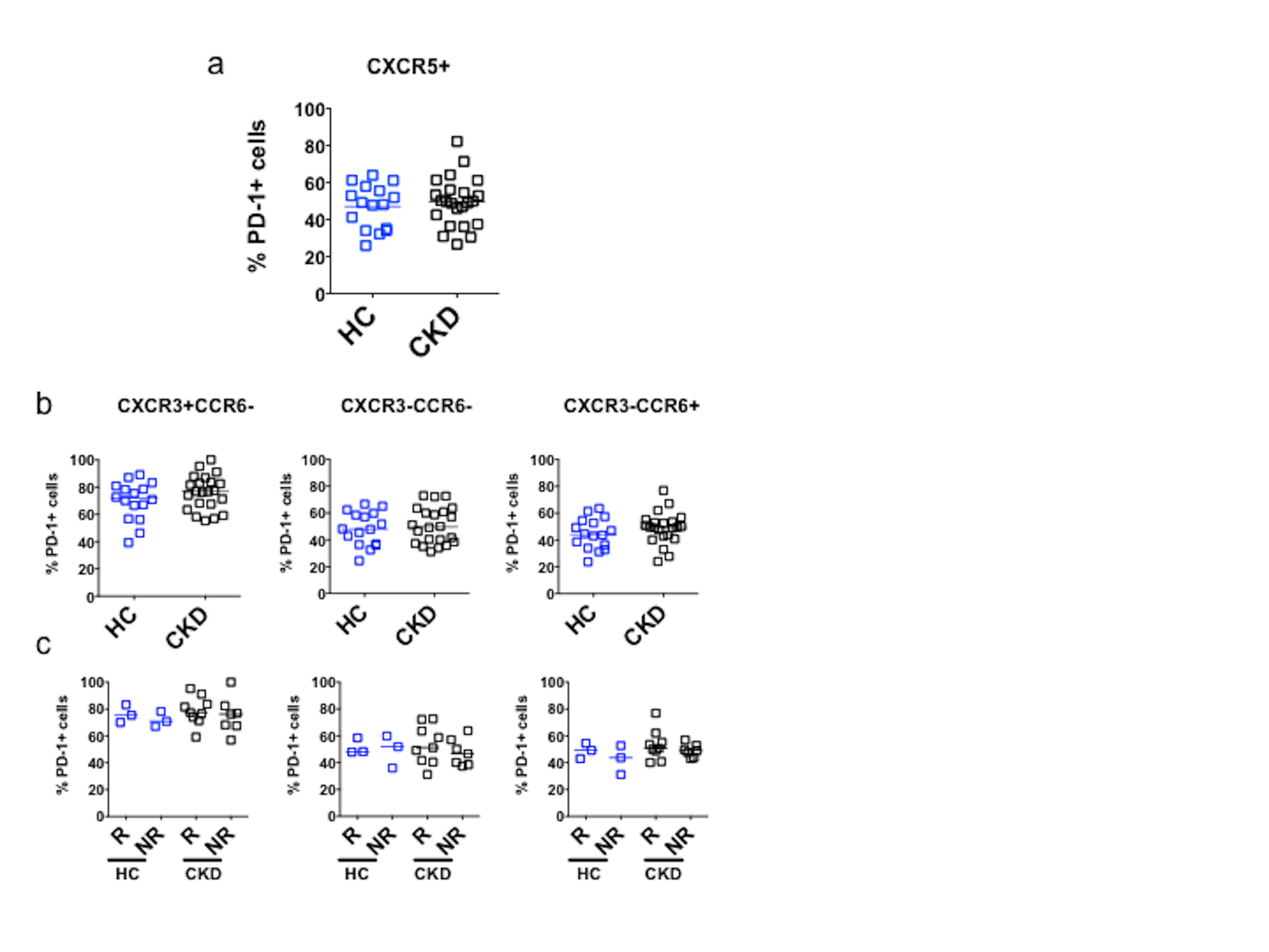

Supplement: S7 Fig — (a). Baseline PD-1+ cells as a percentage of CXCR5+ memory CD4+ T cells. (b). Baseline PD-1+ cells within each cTFH subset, and expressed as a percentage of that subset, in all healthy controls (blue squares, n = 16) and all CKD patients (black squares, n = 22). (c). Baseline PD-1+ cells in cTFH subsets, displayed according to subsequent sero-responsiveness, in healthy controls (HC, blue squares) and CKD patients (CKD, black squares), who subsequently received hepatitis B vaccine and had post vaccination HBsAb measurement performed. Horizontal bars represent medians; analysis performed using Mann-Whitney U test. R–sero-responder (HBsAb ≥10mIU/ml); NR–sero-non-responder (HBsAb <10mIU/ml). (TIF) [file pone.0204477.s007.tif]

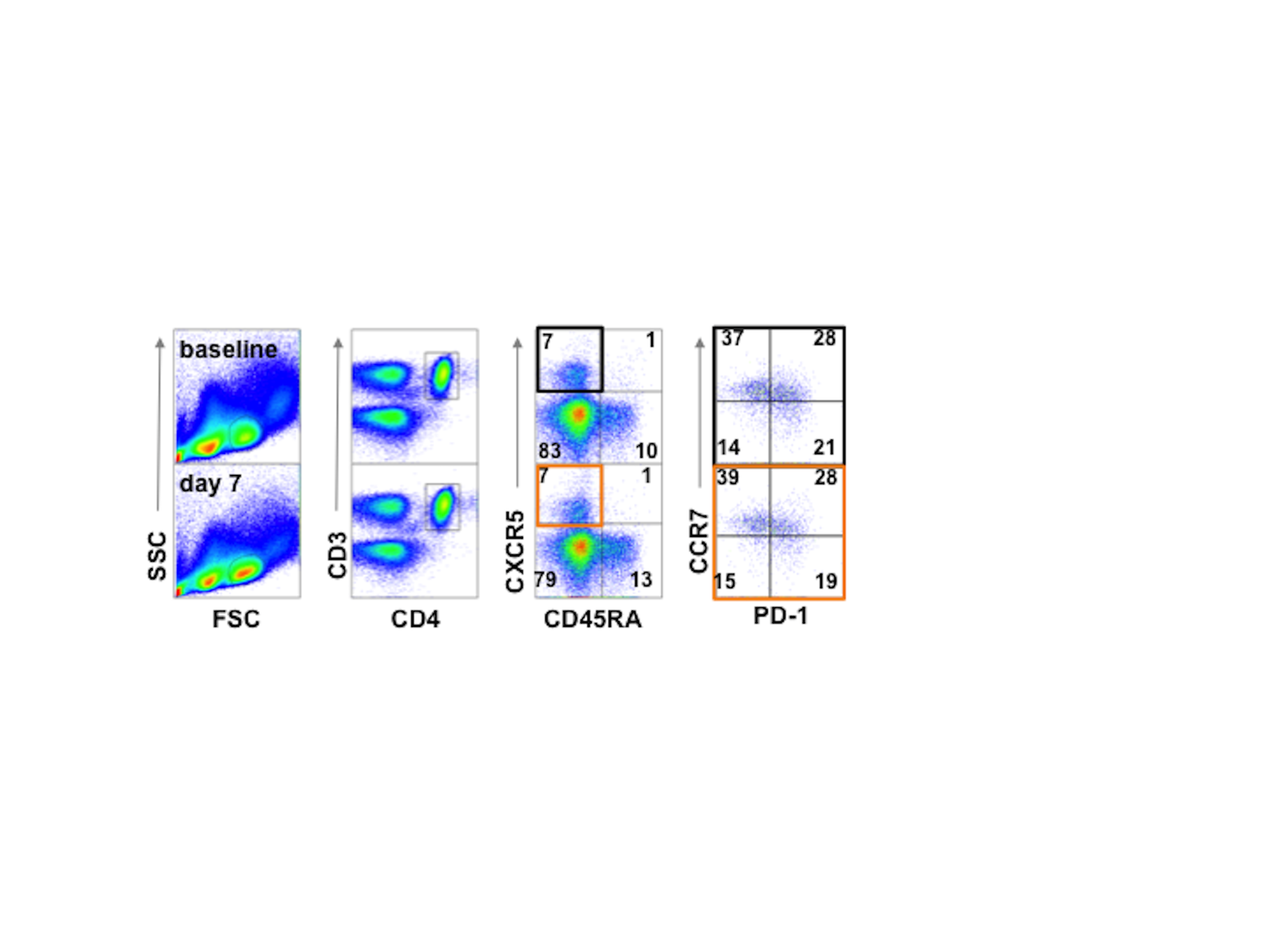

Supplement: S8 Fig — Gating strategy for circulating TFH. After gating on forward and side scatter for lymphocytes, CD3+CD4+CD45RA-CXCR5+ cells were analyzed for expression of PD-1 and CCR7, on samples collected at baseline (before vaccination), and 7 days after vaccination. All values are percentages. (TIF) [file pone.0204477.s008.tif]

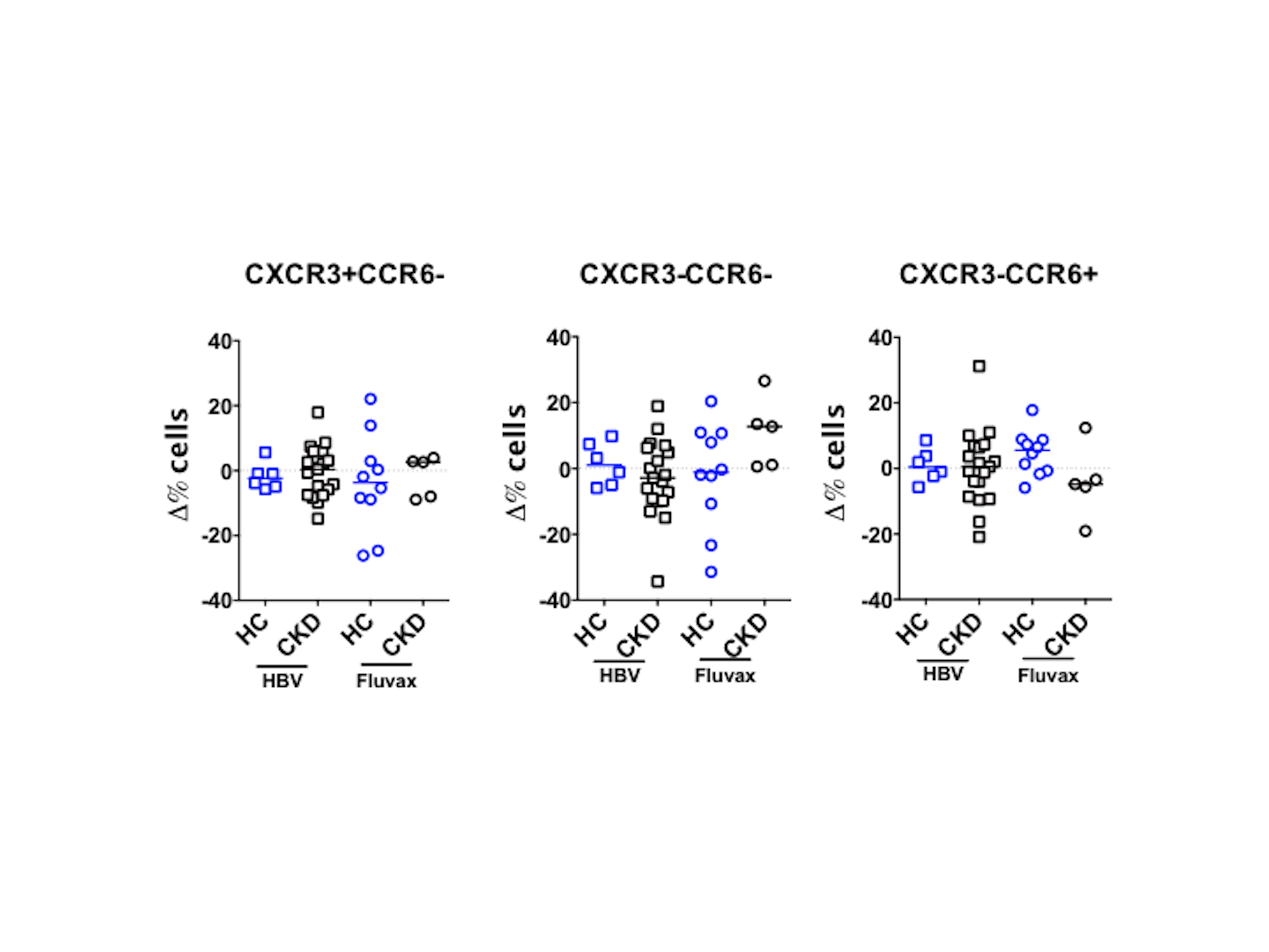

Supplement: S9 Fig — Change (Δ) in TFH subsets (cells as a % of CXCR5+ cells) after vaccination, according to vaccine type and clinical group. HC–healthy controls; CKD–chronic kidney disease; Fluvax–seasonal influenza vaccination; HBV–hepatitis B vaccine. Horizontal bars represent medians. (TIF) [file pone.0204477.s009.tif]

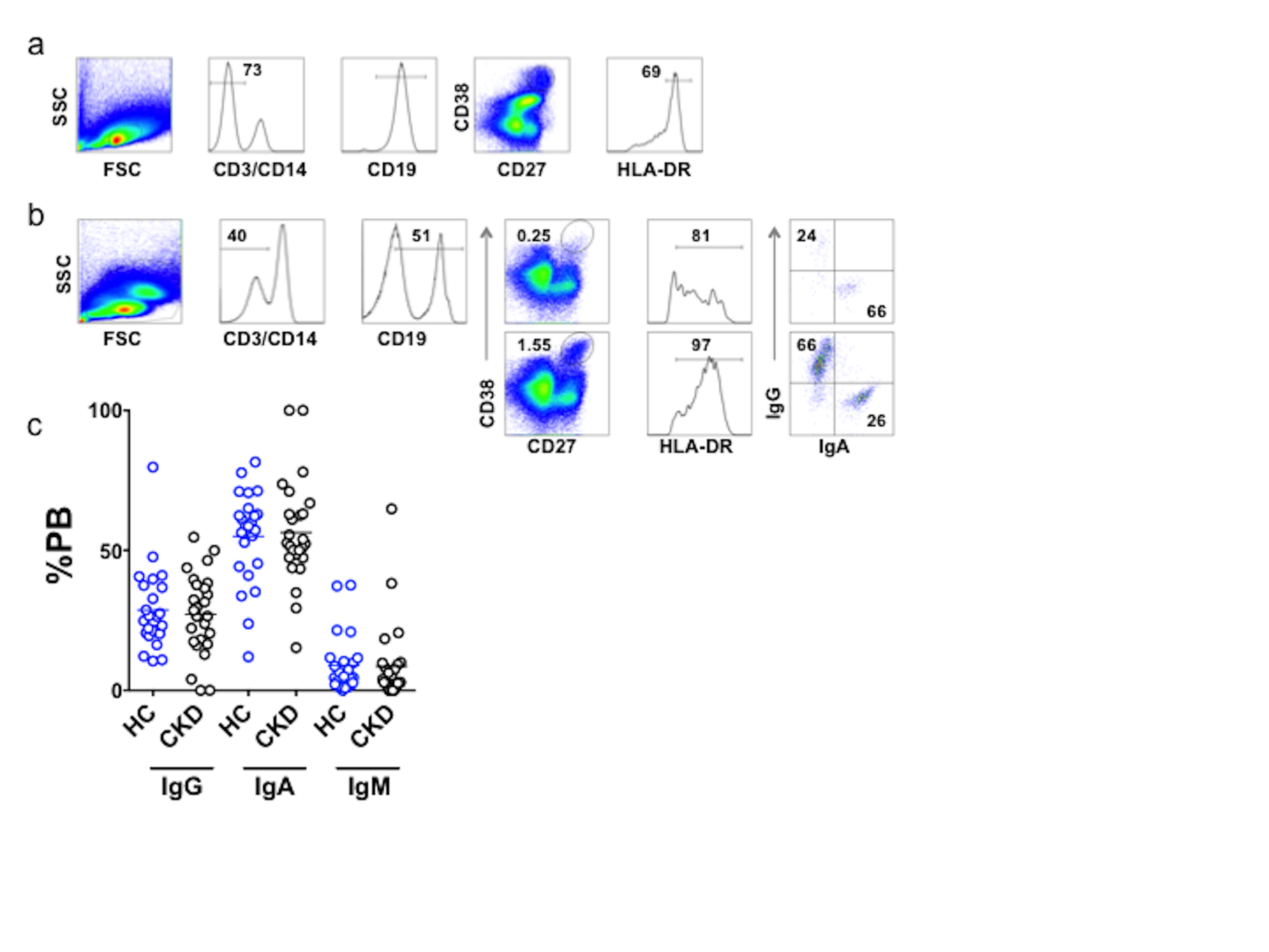

Supplement: S10 Fig — (a) Flow cytometric analysis of human tonsil for the identification of plasmablasts. After gating on lymphocytes according to forward and side scatter, cell populations are selected which are negative for CD3 and CD14 expression, positive for CD19, CD27 and CD38 (oval gate), and express high levels of MHC class II. (b). The same staining and gating strategy is applied to the analysis of peripheral blood. Since plasmablasts downregulate CD19, this gate is set to capture CD19lo cells. Analysis before (top row) and seven days after (bottom row) seasonal influenza vaccination in a healthy control identifies an increase in plasmablasts at day 7, which predominantly express cytoplasmic IgG. (c). Baseline (prevaccination) proportions of plasmablasts according to cytoplasmic immunoglobulin (Ig) isotype expression in healthy controls (HC-blue circles) and CKD patients (black circles); each circles represents an individual. Horizontal bars are medians. PB–plasmablasts. (TIF) [file pone.0204477.s010.tif]

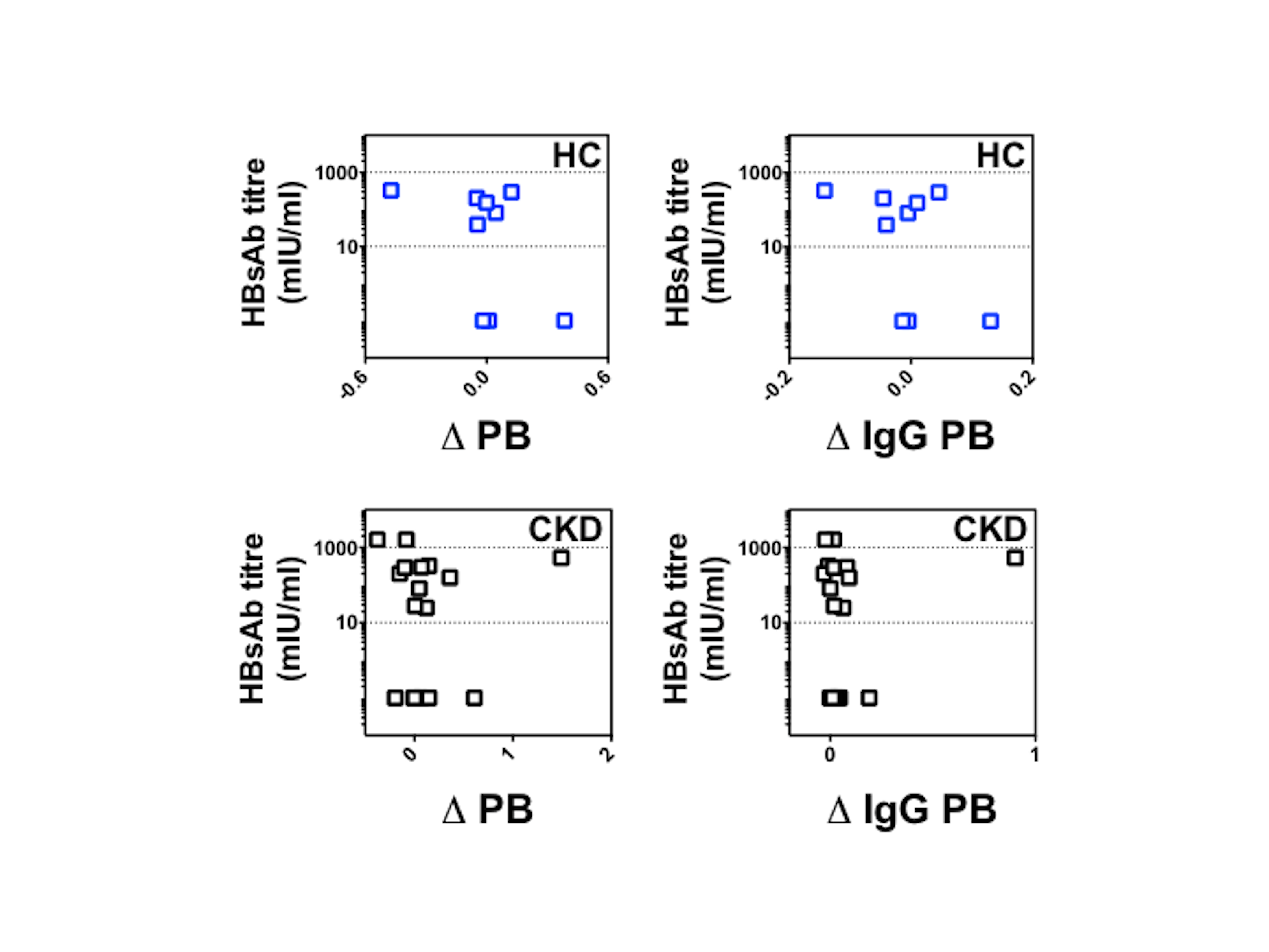

Supplement: S11 Fig — Healthy controls (HC, blue squares) and CKD patients (CKD, black squares); Δ PB—change in total plasmablasts expressed as a percentage of total B cells; (Δ IgG PB) change in IgG plasmablasts expressed as a percentage of total B cells. Dashed horizontal lines represent upper and lower limits of detection of HBsAb assay. HBsAb–hepatitis B surface antibody. Statistical analyses performed using Spearman test of correlation. (TIF) [file pone.0204477.s011.tif]

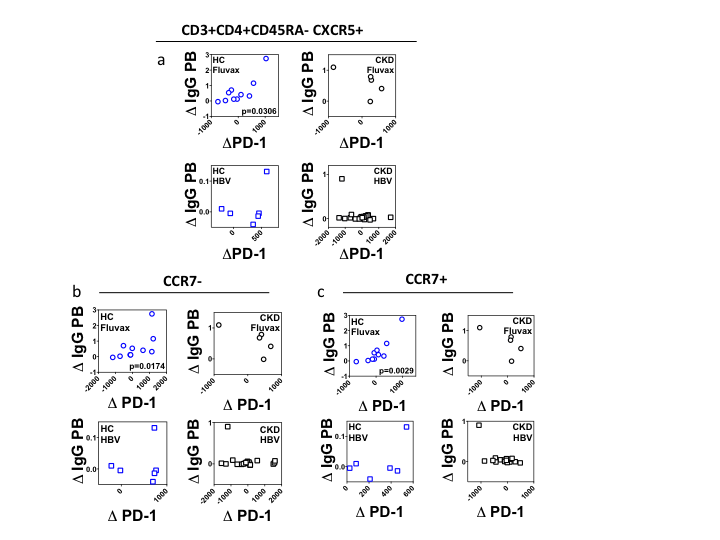

Supplement: S12 Fig — Examination of the relation between change in plasmabalstst (Δ IgG PB) and the change in PD-1 MFI (ΔPD-1) on (a) cTFH, (b) CCR7+ cTFH and (c) CCR7- TFH subsets, according to vaccine group. HC–healthy controls; CKD–chronic kidney disease; Fluvax—seasonal influenza vaccine; HBV—hepatitis B vaccine; HBsAb, -hepatitis B surface antibody. Correlation analysis performed using Spearman test. (TIF) [file pone.0204477.s012.tif]
