# Supplementary material for: A randomized trial of serological and cellular responses to hepatitis B vaccination in chronic kidney disease
Source: PLoS One. 2018 Oct 10;13(10):e0204477. doi: 10.1371/journal.pone.0204477 (PMC6179249; doi:10.1371/journal.pone.0204477)
Supplement: S1 File — (PDF) [file pone.0204477.s013.pdf]

A1 Scientific Project Title : A randomised trial to assess antibody response to hepatitis B vaccine in patients with chronic kidney disease.
(Vaccine CKD study)

A2 Investigators

Dr Krishna Karpe MBBS FRACP (Principal Investigator and contact person)
Renal Unit ; The Canberra Hospital Garran ACT 2605
Email: Krishna.Karpe@act.gov.au
Ph- 02 62442746

Dr Matthew Cook MBBS PhD FRACP FRCPA (Investigator)
Immunology Unit; The Canberra Hospital Garran ACT 2605
Email: Matthew.Cook@act.gov.au
Ph – 02 62445586

Mrs Barbara Harvie MN (Nurse prac) MN (Adv Prac) G Dip Nursing (Neph) (study coordinator and coinvestigator)
Renal Unit ; The Canberra Hospital Garran ACT 2605

[illegible]

Dr Richard Singer MBChB FRACP M Clin Epid (coinvestigator)
Renal Unit ; The Canberra Hospital Garran ACT 2605

Dr Michael Falk MBBS FRACP (coinvestigator)
Renal Unit ; The Canberra Hospital Garran ACT 2605

Dr Giles Walters MBChB MRCP MD FRACP (coinvestigator)
Renal Unit ; The Canberra Hospital Garran ACT 2605

Dr Gavin Carney MBBS FRACP (coinvestigator)
Renal Unit ; The Canberra Hospital Garran ACT 2605

A3. Endorsement by Head of Renal Department

Signature:……………………………………………

Endorsement by Head of Immunology Department

Signature ……………………………………………

Endorsement by CNC Renal Outpatients

Signature……………………………………………

A4. This is not a multicentre study

A5 Plain Language description of the project

Hepatitis B Vaccination (HBV) is recommended for patients with chronic kidney disease. The immune response is reduced and non-sustained in patients with Chronic Kidney Disease (CKD), in particular those with more advanced disease. This study aims to compare augmented HBV vaccination schedule of 40 µg at 0, 1, 2 and 6 months with schedule of 20 µg at 0, 1, 2 and 6 months in patients with stage 4 or 5 Chronic Kidney Disease (CKD). The study also attempts to characterise the immune deficiency of CKD in the cohort in an effort to identify biomarkers that predict vaccine responses.

A 6 Ethical implications of the project

1. Patients will not be enrolled until the trial is reviewed and approved by a Human Research Ethics Committee.

2. Study participants will be recruited by their healthcare professional who will explain to patients that participation is voluntary and non-participation or withdrawal from this research at any time will not prejudice their future care. Only patients capable of providing written informed consent can be enrolled.

3 Protection of patient confidentiality - Patients' records and the data generated by the study will be confidential in line with the recommendations of the NHMRC. Data would be stored in a secure location and Electronic data would have adequate password protection. The participants in this study will be identified only by initials and subject number on these forms.

A7 ***Details of the proposed project***

A7.1 **Background to Project**

Infectious diseases are the second most common cause of death in end-stage kidney disease (ESKD) patients [1]. This may be due, in part, to a relative immunodeficiency associated with chronic kidney disease (CKD). In the case of hepatitis B virus (HBV), patients with CKD are at high-risk of infection from transmission through blood products, contaminated dialysis equipment and cross-contamination from environmental surfaces. The wide application of

universal precautions has reduced the incidence in patients on dialysis but they remain at risk from other environmental exposures. It is been estimated that less than 4% of haemodialysis patients in some developed countries and 10-20% in some developing countries have chronic HBV infections [2]. This is a major health problem world-wide due to the limited availability of effective treatments and high morbidity and mortality. For example, patients with both diabetes and chronic HBV infection are more likely to develop End Stage Renal Disease (ESRD) than those who are not infected [3].

Therefore, prevention of infection through HBV immunisation is recommended for all patients with CKD. The usual HBV vaccination schedule in healthy adults is 20 μg per dose in a three shot schedule (0, 1, 6 months) which results in seroconversion in more than 90% of patients. Unfortunately, the seroconversion immune response is reduced and non-sustained in patients with CKD, in particular those with more advanced disease. For example, a standard HBV vaccination schedule will result in sufficient seroconversion in less than 60% of patients on haemodialysis[4] Therefore, the clinical imperative is to establish an adequate anti-HBs titre in pre-dialysis patients as soon as possible.

Several studies have tried to improve the immune response in patients with ESKD by altering the vaccination schedule. In particular, higher dose (40 μg per dose), additional shots, enhanced immunoreactive adjuvants, co-administration of chemokines or colony stimulating factors and intradermal administration have been trialled.[4, 2] Some studies have shown improved seroconversion to around 80-90% with increased dose and frequency but a definite dose-response relationship was not demonstrated [4]. For example, one randomised controlled trial noted seroconversion in 84% (not statistically significant) of patients with 20 μg at 0, 1, 2, 6 months[5], however a more recent retrospective study noted seroconversion in less than 60% of patients [6]. McNulty et al [7] showed no difference in seroconversion with either 20 or 40 μg per dose in 121 CKD patients. Another study suggested that haemodialysis patients should receive the 80 μg dose due to a prolonged antibody response, despite no improvement in the proportion of patients with seroconversion [8]. Similar outcomes were noted when a new adjuvant was used [9]. Unfortunately, many studies in this area are retrospective or under-powered so the effect on seroconversion can not be confirmed.

There is a lack of consensus for the most effective schedule which should be used for HBV vaccination so a range is used world-wide. For example, the Centers for Disease Control and Prevention in the US recommends 20 μg intramuscularly at 0, 1, 6 months in pre-dialysis patients and 40 μg intramuscularly at 0, 1, 2 and 6 months in dialysis-dependent patients [10]. This would be most rational if there was a discrete change in immune response due to haemodialysis rather than a continuous decline in function, but this has not been confirmed. In contrast, the schedule generally employed in Australia (there are no national guidelines) in patients with CKD is 40 μg intramuscularly at 0, 1 and 6 months. Further, the product information for the HBV vaccine used currently at The Canberra Hospital (Engerix B) recommends a dosing schedule of 40 μg at 0, 1 and 6 months in patients with stage 4 or 5 CKD.

Administration of a 40 μg dose of Engerix B requires two intramuscular injections at each visit, which doubles cost and increases pain. In view of the above variation in vaccine schedules world-wide, the necessity of the 40 μg dose in all pre-dialysis patients with advanced CKD has not been determined. Therefore, more data is required to determine the efficacy of current immunisation schedule for seroconversion to HBV vaccine in patients with CKD.

The mechanisms of impaired humoral immune response in patients with CKD are poorly described. T-cell dependent vaccines such as HBV are particularly affected, which may relate to low levels of CD4+ T-cells and/or T-cell dysfunction due to IL-2 insensitivity [11]. However, it may be multifactorial given that other factors are also associated with immunodeficiency, including macrophage dysfunction, uraemia, obesity, diabetes mellitus, older age, malnutrition and erythropoietin deficiency [12, 4]. Of the few small studies that have been conducted, conflicting results have been obtained. More research into specific measures of B- and T-cell immune function and changes in response to HBV vaccination are required in patients with chronic kidney disease. This may have a role with predicting which patients may require an augmented vaccination regimen. Further, they may have a role as a secondary endpoints in clinical studies assessing the effect of vaccine augmentation.

Patients with CKD show variable response to other vaccines also. From the limited available data, inadequate responses which may require a booster vaccination is noted for varicella,

measles-mumps-rubella and pneumococcus [4]. In contrast, the standard schedule can be used for influenza, Haemophilus influenza type B and inactivated polio virus [4].

It remains to be determined whether an augmented vaccination schedule (increased dose and additional shots) schedule improves seroconversion [12]. Further, clinical and laboratory investigations that predict vaccination failure are required for future research into other methods which may improve seroconversion in patients with CKD.

A7.2 Aims and Hypotheses

Primary objective

To determine whether the augmented HBV vaccination schedule of 40 µg at 0, 1, 2 and 6 months achieves higher rates of seroconversion compared with schedule of 20 µg at 0, 1, 2 and 6 months in patients with stage 4 or 5 CKD.

Secondary objectives

1. The influence of CKD stage as measured by eGFR/1.73m^2 on rates of seroconversion.

2. Characterise the immune deficiency of ESRD in the cohort in an effort to identify biomarkers that predict vaccine responses. In particular, ESRD/HD is associated with lower rates of seroconversion after immunisation with T-dependent antigens, and faster decline in antibody titres among those who seroconvert [13, 14]. These observations raise the possibility of several non-mutually exclusive problems in immunity in ESRD:i. Defect in immune priming as a result of a defect in antigen presentation, or a defect in the primary B cell or T cell repertoire[15]. There is evidence to suggest that the route of vaccine administration affects rates of seroconversion [16], consistent with defects in selective populations of antigen presenting cells.

ii. Defect in T cell or B cell activation .

iii. Defect in clonal expansion.

iv. Defect in terminal B cell differentiation

v. Defect in plasma cell or memory B cell survival

It is established that two broad types of defect in antibody responses occur in patients with ESKD, which are failure of seroconversion, and secondly an unusually rapid decay of serological memory. This suggests the presence of more than one defect in immunity as a result of ESRD. In order to locate the defect, we will evaluate the plasma cell response to vaccination. This will provide an assessment of immune priming, lymphocyte differentiation and clonal explansion. After immunisation, PCs peak in the blood on day 7. Since PCs are normally rare in the blood, virtually all those detectable are antigen-specific. After day 7, PCs numbers in the blood undergo exponential decay as they take up permanent residence in the bone marrow. Second, we will monitor the decay of HBV-specific antibody titres over time.

3. To determine the proportion of patients with a sustained anti-HBs antibody response at 12 months post-vaccination.

A7.3 Methods-This is a randomised prospective trial in patients with CKD. Patients will be randomised into one of two HBV vaccination schedules: schedule A (20μg at 0, 1, 2 and 6 months), or schedule B (40 μg at 0, 1, 2 and 6 months).

Inclusion criteria:

1. Stage 4 or 5 chronic kidney disease
2. Aged over 18 years
3. Treating Physician agreeable to patient's involvement in the trial
4. Informed consent
5. No history of prior hepatitis B infection or hepatitis B vaccination
6. Undetectable anti-HBs antibody on serological testing

Exclusion criteria

1. Current or past hepatitis B infection

2 Detectable anti HBs antibodies.

3. Current treatment with immunosuppressants

4. Known lymphoproliferative disorder

5. Potential non-compliance with treatment regimen in the view of the treating clinicians

6. Involved in another clinical trial where the intervention being trialled is likely to confound the outcome of this trial

7. Previously randomised to this trial.

A7.4 Number of Subjects with statistical validation

To detect a 20% improvement in seroconversion with 80% power, with the two doses and schedule, we need to enrol 81 patients in each group.

A7.5 Methods by which subjects will be recruited

Patients

Renal Medicine at The Canberra Hospital has a database of patients with CKD stages 4 and 5 for which vaccination status has been determined. We have identified more than 200 patients already who require HBV vaccination. Patients who fulfil inclusion and exclusion criteria will be invited to participate in this study when they attend Renal outpatient clinic. Information sheets will be provided regarding the study and if they patient agrees to participate then informed consent will be obtained.

Trial protocol

At entry and before each vaccine dose, all patients would be interviewed and examined. Demographic details, body weight, height, aetiology of CKD (and whether a renal biopsy has been performed) and current drug therapy will be determined. Baseline bloods that are routine for clinical practice will be obtained, including full blood count, clinical chemistry (including creatinine and eGFR) and markers of HBV exposure or vaccination (HBsAg, HBcore antibody and anti HBs antibody). Additional bloods to screen for coexistent immunopathology, including T- and B-lymphocyte subsets, serum immunoglobulins, serum protein electrophoresis, complement and antibody titres to previous vaccinations (tetanus and pneumococcus) will also be obtained.

Allocation to schedule A or B will be conducted using a computer generated set of random numbers.

The HBV vaccine used is Engerix B vaccine 20 μg in 1ml prefilled syringe (recombinant hepatitis B surface antigen adsorbed on aluminium hydroxide adjuvant—SmithKline Beecham, UK). This will be administered intramuscularly in the deltoid muscle using a 23 G × 1″ needle by a registered nurse or medical officer. Patients in schedule A will be administered 20 μg (1mL) and patients in schedule B will be administered 40 μg (2 mL).

Patient follow-up

Patients will be reviewed by a clinician at the time of vaccine administration and according to routine clinical care. At each review the patients will be assessed clinically, weighed and routine clinical bloods will be repeated. Following completion of the schedule at 6 months, seroconversion will be determined six weeks later and followed in each individual after a further 6 months and 12 months. Patients will be withdrawn from the study if their renal function deteriorates to the point that dialysis treatment is needed.

Determination of HBV seroconversion

Anti-HBs antibody titres less than 10 mIU/ml is defined as non-seroconversion. Anti-HBs titres greater than or equal to 10 mIU/ml but less than 100 mIU/ml is defined as seroconversion with low level antibody. Anti-HBs titres greater than or equal to100 mIU/ml is defined as seroconversion with protective levels of hepatitis B antibody.

Enumeration of plasma cells

20mL of peripheral blood will be collected on Day 6/7 after immunisation. We will isolate PBMCs and plasma cells will be enumerated by flow cytometry, according to scatter profiles, and expression of MHC Class II, CD27, CD38 and CD138. Absolute counts will be determined by enumerating lymphocytes on the Coulter counter using an aliquot of the same blood sample.

A7.6 **Estimated duration of study**

The estimated start date for the trial; would be 15th August 2010 and the finish date would be 14/08/2011.

A7.7 **Proposed methods of Data analysis**

Median values of continuous variables would be analysed by Kruskal–Wallis test, and chi-square used to test for association. Multivariable analysis using logistic regression would be employed for eGFR, treatment dose and age as independent variables.

Geometric mean titres (GMTs) of the two vaccine dose groups, derived from the anti-HBs titres obtained at follow-up, will be tested for significance using the two-sample *t*-test.

***A8.* Procedures differing from routine clinical practice/management of patients**

It is a standard practice for all CKD patients to be vaccinated for hepatitis B, however the schedule is augmented which needs additional dose at 2 months, tests for immune markers and repeated measures for anti HBs titers is not routine.

A9. Termination Criteria

The patient withdraws consent.

The patient ceases to be treated at the Canberra Hospital, or is lost to follow-up.

The patient's physician withdraws him/her for medical reasons.

Development of any exclusion criteria.

Non-compliance with study protocol.

A10. Monitoring

Krishna Karpe will monitor the baseline data and anti HBstiters, Barbara Harvie will monitor the patient followup, Matthew Cook will monitor the immunological tests and plasma cell enumeration, Darren Roberts will review the quality of data collection.

A11 Dissemination of results

Who will benefit from the information obtained?

Patients with chronic renal disease and physicians, will benefit from the results of this study as it would indicate the ideal dose schedule and the need if any for augmentation of vaccine in selected population.

Where is it hoped to publish results?

The investigators may publish this information in a peer reviewed journal. All participants will be kept informed of the progress of the study at the conclusion of the trial.

Criteria for authorship -Each author should have participated sufficiently in the work to take public responsibility for the content. Authorship credit should be based only on substantial contributions to (a) conception and design, or analysis and interpretation of data (b) drafting the article or revising it critically for important intellectual content.

A12. Compensation-

Liability insurance has been arranged through the Canberra Hospital Research Insurance scheme.

A13. Report of Project

The Principal Investigator will undertake to provide a statement to the ACTHEC annually and when the research is completed.

A14 See appendix 1

A15.1 See appendix 2

A15.2 The study co-ordinator and study investigators will recruit the subjects and explain the project to the participants if they indicate an interest in participating. The study co-ordinator will act as witness to the informed consent. All participants should be capable of giving their own consent.The subjects will be given a copy of the information sheet and the signed consent form.

References

1. van Dijk PC, Jager KJ, de Charro F *et al.* Renal replacement therapy in Europe: the eesults of a collaborative effort by the ERA-EDTAregistry and six national or regional registries. *Nephrol Dial Transplant* 2001; 16: 1120–1129

2. Unger JK, Peters H. *Hepatitis B in chronic kidney disease: Moving toward effective prevention* Kidney International *(2008) 73, 799–801.*

3. Cheng AY, Kong AP, Wong VW ***et al.*** Chronic hepatitis B viral infection independently predicts renal outcome in type 2 diabetic patients. Diabetologia. 2006 Aug;49(8):1777-84. Epub 2006 May 31.

4. Nicolas Janus, Launay-Vincent Vacher, Svetlana Karie, Elena Ledneva and Gilbert Deray.Vaccination and chronic kidney disease. Nephrol Dial Transplant (2008) 23: 800–807

5. Tong NK, Beran J, Kee SA *et al.* Immunogenicity and safety of an adjuvanted hepatitis B vaccine in pre-hemodialysis and hemodialysis patients. *Kidney Int* 2005; 68: 2298–2303

6. Pin M, Compte MT, Angelet P, Gállego C etal . Long-term evaluation of immune response to hepatitis B vaccine in 136 patients undergoing hemodialysis Nefrologia. 2009;29(5):415-20.

7. McNulty CA, Bowen JK,Williams AJ. Hepatitis B vaccination in predialysis chronic renal failure patients a comparison of two vaccination schedules. *Vaccine* 2005; 23: 4142–4147

8. Chow KM, Law MC, Leung CB, Szeto CC, Li PKT: Antibody response to hepatitis B vaccine in end-stage renal disease patients. Nephron Clin Pract 2006;103:c89–c93.

9. Kong NCT, Beran J, Kee SA *et al.* A new adjuvant improves the immune response to hepatitis B vaccine in hemodialysis patients. *Kidney Int* 2008; 73: 856–862.

10. Centers for Disease Control and Prevention. Recommendations for preventing transmission of infections among chronic hemodialysis patients. *June 2006*

11. Litjens NH, Huisman M, Baan CC, van Druningen CJ, Betjes MG: Hepatitis B vaccine-specific CD4(_) T cells can be detected and characterised at the single cell level: Limited usefulness of dendritic cells as signal enhancers. *J Immunol Methods* 330: 1–11, 2008

12. Uzi Gafter How to Improve Anti-Hepatitis B Vaccination Nephron Clin Pract 2006;103:c87–c88

13. Tsouchnikas I, Dounousi E, Xanthopoulou K, Papakonstantinou S, Thomoglou V, Tsakiris D.Loss of hepatitis B immunity in hemodialysis patients acquired either naturally or after vaccinationClin Nephrol. 2007 Oct;68(4):228-34.

14. Peces R, Laurés AS. Persistence of immunologic memory in long-term hemodialysis patients and healthcare workers given hepatitis B vaccine: role of a booster dose on antibody response. Nephron. 2001 Oct;89(2):172-6.

15. Pahl MV, Gollapudi S, Sepassi L et al. Effect of end-stage renal disease on B-lymphocyte subpopulations, IL-7, BAFF and BAFF receptor expression. Nephrol Dial Transplant. 2010 Jan;25(1):205-12. Epub 2009 Aug.

16. Barraclough KA, Wiggins KJ, Hawley CMet al. Intradermal versus intramuscular hepatitis B vaccination in hemodialysis patients: a prospective open-label randomized controlled trial in nonresponders to primary vaccination. Am J Kidney Dis. 2009 Jul;54(1):95-103. Epub 2009 May 29.

PATIENT INFORMATION SHEET

A randomised trial to assess antibody response to hepatitis B vaccine in patients with chronic kidney disease.

Research study:

This document may help you to understand the information regarding your participation in a clinical research study. It may assist you in deciding whether or not to take part- after knowing the possible risks and benefits of such a study. The information obtained from this study may possibly be helpful to others.

Purpose of the study:

The purpose of the study is to compare augmented Hepatitis B vaccination (HBV) schedule of 40 µg at 0, 1, 2 and 6 months with schedule of 20 µg at 0, 1, 2 and 6 months in patients with stage 4 or 5 Chronic Kidney Disease (CKD). The study also attempts to characterise the immune deficiency of CKD in an effort to predict vaccine responses.

Why is the study being performed?

Prevention of infection through Hepatitis B Vaccination (HBV) is recommended for all patients with Chronic kidney disease (CKD). The usual HBV vaccination schedule in healthy adults is 20 µg per dose in a three shot schedule (0, 1, 6 months) which results in seroconversion in more than 90% of patients. Unfortunately, the seroconversion response is reduced and non-sustained in patients with CKD.

There is a lack of consensus for the most effective schedule which should be used for HBV vaccination and patients with CKD also show variable response to vaccines. More research into specific measures of immune response to vaccination are required in patients with chronic kidney disease.

Who will be asked to enter the study?

You will be invited to participate in the trial if you have stage 4 or 5 Chronic kidney disease.

What will happen on this study?

If you choose to participate, you will be either placed in the vaccine schedule A or schedule B based on a group of random numbers. Indivisuals in schedule A would be vaccinated with 20 mcg of the

vaccine as opposed to 40 mcg dose in schedule B. Blood tests would be obtained for testing on entry to the study and subsequently to determine response to the vaccine. No other aspect of your clinical care is altered. You will still be looked after by your usual health care team. All data will be recorded on a data collection sheet.

Tests	Baseline	Day 7 post first vaccine dose	4-6 weeks after Completion of vaccination	6 months post vaccination	12 months post vaccination
History	X				
Examination	X				
Anti HBs titres	X		X	X	X
Other blood tests	X	X	X	X	X

Are there any risks?

There is a large amount of clinical experience with the use of. It has also been shown to prevent infections. No serious side effects have been reported to date. Nevertheless, it is possible that skin irritation or other, as yet unreported, side effects may occur.

Potential Benefits

If you decide to participate in the trial, your condition will be very closely monitored. Any changes in your condition will be very quickly identified. The results of the study may also provide information, regarding the ideal dose and schedule for vaccination in indivisuals with chronic kidney disease.

Confidentiality

All information gained from this study will be treated with utmost confidentiality without reference to your name. You will be asked to give permission to any regulatory authorities related to the study to access your medical records. The information will only be used for purposes of this study. Medical records will be stored for accessibility for at least 15 years.

Costs

Your participation in this study will not cost you any money. You will not receive any payment for participation in this study. Participation in this study will not change usual hospital liability arrangements and processes.

Do you have a choice?

Entry into the study is voluntary. Choosing not to take part in this study will not affect your treatment or care in any way. The doctors will continue to treat you with the best means available.

If you agree to participate in this study you will be asked to sign a consent form. However you may withdraw from the study at any time without giving a reason. You will be told if any new information arises which might affect your decision to be in the study.

Ethics Approval

This study has been reviewed and approved by the local Ethics Committee. Should you wish to discuss the matter with someone not directly involved, you can contact the ACT Health Human Research Ethics Committee Secretary at the ACT Health Research Office, Canberra Hospital, Yamba Drive, Garran, ACT 2605 or by telephone on (02) 6205-0846.

Consent Form to Participate in a Research Project

I, ____

(name of participant)

of ____

(street) (suburb/town) (state & postcode)

have been asked to consent to my participation in a research project entitled: A randomised trial to assess antibody response to hepatitis B vaccine in patients with chronic kidney disease.

In relation to this project I have read the Patient Information Sheet and have been informed of the following points:

1. Approval has been given by the ACT Department of Health Ethics Committee.

2. The aim of the project is to compare two schedules of Hepatitis B vaccination in Chronic Kidney Disease and attempt to characterise the immune deficiency in an effort to predict vaccine response.

3. The drug is available for use in Australia.

4. The results obtained from the study may or may not be of direct benefit to my medical management.

5. The procedure will involve vaccination against hepatitis B.

6. My involvement in this project may be terminated if at any time I decide to withdraw from the trial, OR my physician decides that I withdraw due to adverse effects.

8. Should I develop a problem which I suspect may have resulted from my involvement in this project, I am aware that I may contact the Mrs Babara Harvie or Dr Krishna Karpe,

9. Should I have any problems or queries about the way in which the study was conducted, and I do not feel comfortable contacting the research staff, I am aware that I may contact the ACT Department of Health Ethics Committee Secretary on 02-62050846.

10. I can refuse to take part in this project or withdraw from it at any time without affecting my medical care.

11. Participation in this project will not result in any extra medical and hospital costs to me.

12. I understand that the results of the research will be made accessible and that my involvement and my identity will not be revealed.

13. In giving my consent, I acknowledge that the relevant clinical staff directly involved in the study, may examine my medical records only as they relate to this project.

After considering all these points, I accept the invitation to participate in this project. I also state that I have/have not participated in any other research project in the past 3 months. If I have, the details are as follows:

Date: ________________ Participant:

____

(Please print name) (Signature)

Date: ________________ Witness:

____

(Please print name) (Signature)

Investigator's Signature:

____

Section B – BUDGET

B1. Study Details

B1.1 Project Title

A randomised trial to assess antibody response to hepatitis B vaccine in patients with chronic kidney disease

B1.2 Chief Investigator Dr Krishna Karpe

B2 Income

B2.1 Source of Funding The Canberra Hospital Private Practice Resaerch fund (pending approval)

B2.2 Details of Funding

Total – & 13783.20

TCH Cost Centre from which funding will be administered

___**To be announced**______________________

____

(Fund source/cost centre) (Name of account)

B3 Consultancy Fees and Additional Payments

B4 Expenditure

B3.1 Personnel – specifically salaried or part salaried for this project

Nil

B4.2 Personnel – not employed specifically for this project

Nil

B4.3 Personnel – Honorary

Position	2 Specialists
Hours/Month	8 hours/month each

B4.4 Estimated Service Costs

Pathology costs - $ 6520

B4.5 Administrative Costs

Total $500

B4.6 Data Handling/Computing

Nil

B4.7 Patient/Participant Costs
Total $500

B4.8 Travel (study related for investigators and/or participants)

Nil

B4.9 Equipment

pneumococcal antibody kit - $ 3950

Vaccine costs - $2203.20

B4.10 General Supplies and Consumables

Nil

B4.11 Other Costs (eg payments to volunteers)

Nil

B4.12 Ethics Committee Fee

Total $ 110

B4.13 Total Expenditure

Total Expenditure $ **$13783.2**

B4.14 Difference between total expenditure and total income is

T1-T2 = $ 0 Surplus

B4.16 Duration of the Study

Years/Months 12 months

Start Date 15th August 2010

End Date 14th August 2011
